# Trends in HIV self-testing uptake in Africa: A modeling study of population-based surveys and HIV testing program data

**DOI:** 10.1371/journal.pmed.1004771

**Published:** 2026-05-05

**Authors:** Aishi Aratrika, Carla M. Doyle, Cheryl Case Johnson, Olanrewaju Edun, Bogol Mbope, Achille Adoko, Mphotleng Tlhomola, Cédric P. Yansouni, Augustine Talumba Choko, Jeffrey W. Imai-Eaton, Mathieu Maheu-Giroux

**Affiliations:** 1 Department of Epidemiology and Biostatistics, McGill University, Montréal, Québec, Canada; 2 Department of Public Health, North South University, Dhaka, Bangladesh; 3 HIV, Tuberculosis, Hepatitis and STIs Department, World Health Organization, Geneva, Switzerland; 4 MRC Centre for Global Infectious Disease Analysis, School of Public Health, Imperial College London, London, United Kingdom; 5 Ministry of Health, Programme national de lutte contre le Sida (PNLS), Kinshasa, Democratic Republic of the Congo; 6 The Joint United Nations Program on HIV/AIDS (UNAIDS) Country Office, Cotonou, Benin; 7 Ministry of Health, Maseru, Lesotho; 8 JD MacLean Centre for Tropical and Geographic Medicine, and Divisions of Infectious Diseases and Medical Microbiology, McGill University Health Centre, Montréal, Québec, Canada; 9 Malawi Liverpool Wellcome Trust Clinical Research Programme, Blantyre, Malawi; 10 Center for Communicable Disease Dynamics, Department of Epidemiology, Harvard T.H. Chan School of Public Health, Harvard University, Boston, Massachusetts, United States of America; University of Washington Department of Global Health, UNITED STATES OF AMERICA

## Abstract

**Background:**

HIV self-testing (HIVST) can increase access to and uptake of HIV testing among people underserved by other HIV testing approaches. Several countries in Africa, the region most affected by HIV, have scaled-up HIVST. However, no comprehensive analysis has yet quantified HIVST uptake trends and how HIVST kits are used. We aimed to estimate 1) country-level and regional trends in HIVST uptake among adults by sex and age and 2) the proportion of distributed HIVST kits that are used and re-testing rates with HIVST.

**Methods and findings:**

Across African countries, we analyzed 1) data from national population-based surveys that included questions on previous HIVST use and 2) the number of HIVST kits distributed from nationally reported program data (2012–2024). We developed a hierarchical Bayesian compartmental model to estimate HIVST rates by triangulating surveys and program data. Random effects were used to pool information across countries. Data were available from 40 surveys in 27 countries and from 99 country-years of HIVST program data. The proportion of adults aged ≥15 years in Africa who have ever used an HIVST (HIVST uptake) steadily increased, from <1% in 2012 to almost 7% (6.8%; 95% credible interval [95%CrI] [5.8, 8.2]) in 2024. HIVST uptake was higher in eastern and southern Africa (10.2% in 2024, 95%CrI [8.5, 12.7]) compared to western and central Africa (2% in 2024; 95%CrI [1.7, 2.5]). The proportion of people who ever self-tested varied substantially across countries, reaching a maximum in 2024 of 45.4% (95%CrI [41.8, 51.5]) in Lesotho. Men (7.2% in 2024, 95%CrI [6.1, 8.8]) were slightly more likely than women to have ever used an HIVST (6.4% in 2024; 95%CrI [5.4, 7.8]). Compared to younger individuals (15–24 years), those aged 25–34 years had higher rates of self-testing (men: rate ratio [RR]=1.8, 95%CrI [1.5, 2.3]; women: RR = 1.4, 95%CrI [1.1, 1.6]). Individuals who previously self-tested may be more likely to self-test again (RR = 1.1, 95%CrI [0.8, 1.5]), although with substantial uncertainty. We estimated that 70% (95%CrI [60, 80]) of all HIVST distributed were used. Limitations of the study include challenges in precisely estimating some parameters, exclusion of countries without any HIVST distribution data and inability to model HIVST positivity.

**Conclusions:**

HIVST uptake has increased in Africa, with wide variation between countries. HIVST is more likely to engage 25–34-year-olds and men, who have historically been less likely to be aware of their HIV status. Our results can help understand patterns of use and support countries in optimizing their HIV testing services.

## Author summary

**Why was this study done?:**

- Despite major progress in expanding access to HIV testing services (HTS) over the past decades, substantial gaps in diagnosis coverage persist, particularly among men and key populations in Africa.
- HIV self-testing (HIVST) offers a private, acceptable, and cost-effective option to overcome persistent barriers to testing access and uptake.
- However, monitoring uptake remains difficult, as routine program data captures only kit distribution, not actual utilization or user outcomes.
- We aimed to estimate regional and country-specific trends in HIVST uptake, disaggregated by sex and age, and to understand how HIVST kits are used (re-testing, percentage of distributed kits used) to guide future HTS strategies.

**What did the researchers do and find?:**

- We developed a mathematical model to estimate HIVST uptake from 2012 to 2024 among adults aged ≥15 years across Africa. We triangulated two data sources (2012–2024): population-based surveys on the proportion of HIVST use and national program data on the number of HIVST kits distributed. Data included 40 surveys from 27 countries and 99 country-years of HIVST program data.
- HIVST uptake increased steadily across Africa, with large regional differences: much higher in eastern and southern Africa compared to western and central Africa.
- Uptake varied widely by country, reaching 45% in 2024 in Lesotho. Men were slightly more likely than women to have ever self-tested. Compared with 15–24-year-olds, self-testing rates were higher among 25–34-year-olds, followed by 35–49-year-olds and those 50+. Additionally, we estimated that most of distributed HIVST kits were likely used.

**What do these findings mean?:**

- HIVST is now an important testing modality in Africa, with evidence of rising uptake in many countries, especially among men and adults aged 25–34-year-olds.
- These patterns suggest HIVST can help close remaining diagnosis gaps. Our findings support continued maintenance and expansion of HIVST within national HTS strategies.
- A limitation of this study is the lack of direct data about re-testing rates and fraction of kits used.

## Abstract 3

Using population-based survey and program data for from 27 countries in Africa, Aishi Aratrika and colleagues generate estimates of uptake of HIV self-testing to help inform country needs and optimize HIV testing services.

## Introduction

HIV testing services (HTS) are essential for reaching people living with HIV (PLHIV) who do not know their status and are a critical entry point to both HIV prevention and treatment services. Despite the substantial success scaling-up HTS over the last three decades, gaps in testing coverage remain [1]. HIV self-testing (HIVST), whereby an individual performs a blood-based or oral rapid test and interprets the result on their own in private settings, has been shown to be an important tool that can help address remaining gaps by increasing access to and uptake of HIV testing [2,3].

Africa remains disproportionately affected by HIV, home to 26 million PLHIV and experiencing an estimated 640,000 new HIV acquisitions in 2023 [4]. To end the HIV epidemic, the *Global AIDS Strategy* has set targets for 95% of PLHIV to be aware of their status, 95% of diagnosed PLHIV on treatment, and 95% of treated PLHIV to be virally suppressed by 2030 [5]. Countries in eastern and southern Africa are closer (around 93% PLHIV being aware of their status in 2023) to achieving this 95% diagnosis coverage target, compared to 81% of PLHIV in western and central Africa [6,7].

Progress toward the first 95 target is hampered by persistent and multifaceted barriers to HIV testing, often disproportionately impacting adult men and key populations. Such barriers include differential engagement with health services that provide less entry points for testing, internalized and anticipated stigma, discrimination, criminalization, punitive and restrictive laws, privacy concerns, gender norms, poverty, and geographical inaccessibility [8–16]. While traditional provider-delivered HTS approaches in antenatal care, outpatient settings and focused community outreach have been impactful, closing the remaining gaps will require a more diverse and expanded package of HTS options, particularly for populations with undiagnosed PLHIV who are not accessing services [3,17,18].

HIVST is a safe, acceptable, and discreet HIV testing option to overcome some of the persistent barriers to service delivery. The *World Health Organization* (WHO) first recommended HIVST in 2016 [3,8,19]. Two large scale multi-country initiatives, the *STAR (HIV Self-Test Africa)* Initiative launched in 2015 and the *ATLAS (Auto Test VIH, Libre d’Accéder à la connaissance de son Statut)* launched in 2018, contributed to expanding self-testing and HIV diagnosis in distinct epidemiological contexts [20,21]. Mathematical modeling and economic evaluations based on these programs demonstrated that HIVST improve treatment coverage, reduce HIV incidence, and is consistently cost-effective [22,23]. Routinely monitoring HIVST uptake and its subsequent effects on strengthening HIV diagnosis is challenging, however. Program data typically capture only the number of HIVST kits distributed, without information on actual use, user characteristics, confirmed test results, or onward linkage to further testing, treatment, or prevention services. Disclosure of reactive self-test results may not be consistently reported or recorded at the individual or program level. Population-based surveys collecting self-reported information on awareness and use of HIVST can be useful to track uptake, but are conducted at 5-year intervals in the best of cases. Consequently, estimates of HIVST uptake remain limited to country-specific, small-scale studies on HIVST trends [24–28].

To improve our understanding of HIVST uptake, we synthesized self-reported HIVST uptake data from population-based surveys and annual self-test kit distribution data across African countries. We developed a hierarchical mathematical model to triangulate these data sources and generate country and regional estimates of HIVST uptake by sex and adult age groups. We also estimated HIVST re-testing rates and the proportion of HIVST kits that were likely used from the model.

## Methods

### Data sources

We collated data from two sources: *(1)* nationally representative population-based surveys that included questions on participants’ self-reported previous use of HIVST and *(2)* country-level HIV testing program data on the annual number of HIVST kits distributed. To identify eligible surveys, we reviewed all publicly available, cross-sectional, population-based surveys from countries across eastern, southern, central, and western Africa conducted between January 2012 and December 2024. The earliest date corresponds to the first survey to include self-reported HIVST uptake information, which was the 2012 *Kenya AIDS Indicator Survey* (KAIS) [29]. We systematically searched data catalogs (i.e*., the Global Health Data Exchange, WHO Multi-Country Studies Data Archive*) and conducted Google engine and literature searches. We identified the surveys with HIVST usage-related items, often measured through the question ‘*Have you ever tested yourself for HIV using a self-test kit?’* (see Table A in S1 Appendix for the list of all survey questions). The following survey series were identified: *Demographic and Health Surveys* (DHS), *AIDS Indicator Cluster Surveys* (AIS), *Multiple Indicator Cluster Surveys* (MICS), *Population-based HIV Impact Assessments* (PHIA), *Botswana AIDS Impact Survey* (BAIS), and KAIS. For each survey, we calculated the sex- and age-stratified proportions of respondents aged ≥15 years who reported ever using an HIVST, applying the survey weights accounting for the multi-stage cluster sampling designs.

We collected information on the annual number of HIVST kits distributed between January 2016 and December 2024 from multiple sources: spectrum model files submitted by national HIV programs to UNAIDS for the 2024 national HIV estimates round, WHO*/UNAIDS Global AIDS Monitoring* (GAM) system (2018–2023), the 2024 *PANAROMA* dataset of the *President’s Emergency Plan for AIDS Relief* (PEPFAR), and national HIVST procurement records reported to WHO (2016–2024). For both Spectrum and GAM, HIV testing data are routinely reported jointly to WHO and the UNAIDS by countries through their national HIV testing programs. Where data for the same country-year were available from multiple sources, we prioritized data in the following order: *1)* Spectrum estimates files, *2)* GAM, or *3)* PEPFAR because Spectrum allows countries to update or revise previously submitted data in subsequent years, whereas data submitted to GAM are fixed once reported and cannot be modified retrospectively if found to be incorrect.

Our analysis considered countries with at least one available survey and one year of HIVST distribution data, as these were the minimum data required to calibrate the model.

### Mathematical modeling of HIVST uptake

**Fig 1:**
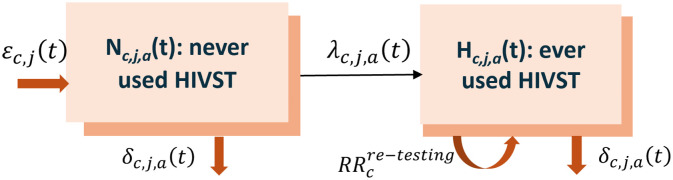
Diagram of the compartmental flows of the deterministic model of HIV self-test (HIVST) users and nonusers. Indices: c = country, j = sex, a = age group (15-24, 25-34, 35-49, ≥ 50), t = year (2012–2024). $\varepsilon_{c,j}(t)$: yearly entry rate of 15–24-year-olds; $\delta_{c,j,a}(t)$: annual death rate; $\lambda_{c,j,a}(t)$: annual HIV self-testing rate; $\overset{re-testing}{\underset{c}{RR}}$: HIV self-testing re-testing rate ratio.

We developed a deterministic compartmental model to estimate HIVST uptake over 2012–2024 among open populations of individuals aged ≥15 years based on a system of ordinary differential equations (Fig 1). The model’s structure was chosen to match the survey (i.e., having ever used an HIVST) and program data (i.e., annual number of HIVST kits distributed) available to inform it, and adapted from a previous model of HIV testing behaviors [1,30]. The model partitioned the population into individuals who have never used an HIVST (*N*) and individuals who have ever used an HIVST (*H*). We further stratified the model by sex and age groups (15–24, 25–34, 35–49, and ≥50 years). Entry into the model occurs in the never HIVST compartment for individuals reaching age 15 years and individuals exit the model through death. Demographic inputs were sourced from the United Nations 2024 *World Population Prospects* (WPP) Revision [31].

All individuals were assumed to have never used an HIVST at model initiation in 2012. People can use HIVST at a time-varying rate ($\lambda_{c,j,a}(t)$) which was modeled as a first-order random walk with annual time steps (*t*). These self-testing rates are country-specific (*c*) and vary by sex (*j*) and age groups (*a*). Individuals who previously used HIVST may be more or less likely to use it again, depending on how HIVST distribution is implemented (e.g., HIVST distributed to key populations). To account for that, we introduced a re-testing rate ratio (RR) for people in the ever self-tested (*H*) compartment.

The model parameters’ prior distributions are presented in Table 1. Our main assumptions, differential equations, and other additional details can be found in Text A (S1 Appendix).

**Table 1:** List of main parameters, their definitions, prior distributions, and justifications for the mathematical model of HIV self-testing uptake.

Parameters	Definition	Prior	Justification
$log(\tau_{c}\left(\text{1})\right)$	Baseline country-specific (c) testing rate for females aged 15–24 (i.e., the referent group)	~N(−10, 1)	Log-normal distribution. Almost zero self-testing initially. Prior density: 0.000045 per year (0.000006–0.00033).
$log(\tau_{c}\left(t>\text{1})\right)$	Country-specific (c) testing rates for subsequent years for females aged 15–24 (i.e., the referent group)	$\sim N(log(\tau_{c}(t-\text{1})),\;\sigma_{RW})$	HIVST testing rates are modeled (log-scale) as a first-order random walk (RW1) process.
$\sigma_{RW\;}$	Standard deviation of the first-order random walk (RW1) process	~N(0, 0.25)	Weakly informative truncated normal distribution [0.000001, 5].
*log(* $\overset{m-to-f}{\underset{overall}{RR}}\;)$	Overall 15–24-year-old male-to-female HIVST rate ratio	~ N(0, 0.5)	No overall difference in HIVST rates is assumed between males and females aged 15–24. Overall male-to-female ratio is 1.0 with 95% of the prior density between 0.38 and 2.66 approximately.
$\overset{male}{\underset{c,j}{log(RR}})$	Country-specific (c) 15–24-year- old male-to-female HIVST rate ratio	Derived from:$log(\overset{m-to-f}{\underset{overall}{RR}})+\sigma_{{RR}_{m-to-f}}\;\overset{m-to-f}{\underset{c}{RRn}}$ where$\overset{m-to-f}{\underset{c}{RRn}}\;\sim \;N(\text{0},\text{1})$	$\overset{m-to-f}{\underset{c}{RRn}}\;$is the standard normal variable for country *c.* A typical country is expected to have a male-to-female HIVST rate ratio between ~0.38 and 2.66, centered around the overall mean of 1.0.
$\sigma_{{RR}_{male}}$	Between-group standard deviation of country-specific 15–24-year- old male-to-female HIVST rate ratio	~N(0, 0.5)	Weakly informative truncated normal distribution [0.000001, 5].
$log\left(\overset{age}{\underset{a,j}{RRoverall}}\right)$	Overall age-specific HIVST rate ratios for age group *a* and sex *j*	~ N(0, 0.5)	No difference is assumed across sexes and age groups at baseline (centered at 1.0), while allowing flexible variation. 95% of prior density lies between 0.38 and 2.66.
$log(\overset{age}{\underset{c,a,j}{RR}})$	Country-specific (c) rate ratios by group *a* and sex *j*	Derived from: $log(\overset{age}{\underset{a,j}{RRoverall}})+\;\sigma_{\overset{age}{\underset{a}{RR}}}\overset{age}{\underset{c,a,j}{RRn}}$where, $\overset{age}{\underset{c,a,j}{RRn}}\;\sim \;N(\text{0},\text{1})$	$\overset{age}{\underset{c,a,j}{RRn}}\;$is the standard normal variable for country *c,* age group *a*, and sex *j.* Country-specific deviations are modeled around the overall age effect on the log-scale. 95% of country-specific rate ratios by age and sex are expected to fall between ~0.38 and 2.66.
$\sigma_{\overset{age}{\underset{a}{RR}}}$	Between-group standard deviation of country-specific rate ratios by age and sex	~N(0, 0.5)	Weakly informative truncated normal distribution [0.000001, 5].
${RRoverall}^{re-testing}$	Overall HIVST re-testing ratio (logit scale)	$\sim N(logit(\frac{\text{1}.\text{2}\;-\;\text{0}.\text{5}}{\text{2}.\text{5}\;-\;\text{0}.\text{5}}),\;\text{0}.\text{5})$	Mean overall re-testing ratio is 1.2, with 95% of the prior density between 0.84 and 1.68 [33,34].
$\overset{re-testing}{\underset{c}{RR}}\;$	Country-specific (c) HIVST re-testing ratio	Derived from: $\text{0}.\text{5}+\text{2}\;{logit}^{-\text{1}}\left({RRoverall}^{re-testing}+\overset{re-testing}{\underset{c}{RRn}}\;\sigma_{{RR}_{rt}}\right)$ where:$\overset{re-testing}{\underset{c}{RRn}}\sim \;N(\text{0},\text{1})$	$\overset{re-testing}{\underset{c}{RRn}}$: is the standard normal variable for country *c.* Typical countries are expected to have a re-testing rate ratio between 0.84 and 1.68, centered around the overall mean of 1.2, with variation driven by country-specific effects.
$\sigma_{{RR}^{re-testing}}$	Between-group standard deviation of country-specific HIVST re-testing ratio	~N(0, 0.5)	Weakly informative truncated normal distribution [0.000001, 5].
$\phi_{overall}$	Overall proportion of distributed HIVST kits used (logit scale)	$\sim N(logit(\frac{\text{0}.\text{8}\text{5}-\text{0}.\text{5}}{\text{1}\;-\;\text{0}.\text{5}}),\;\text{0}.\text{5})$	Overall proportion of HIVST kits used is centered around 85%, with 95% of the prior density between ~63% and 97% [35].
$\phi_{c}$	Country-specific (c) proportion of distributed HIVST kits used	Derived from: $\phi_{c}=\;\text{0}.\text{5}+\text{0}.\text{5}\;{logit}^{-\text{1}}\left(\phi_{overall}+\phi_{raw}\;\sigma_{\phi \;}\right)\;$where:$\phi_{raw}\;\sim \;N(\text{0},\text{1})$	Each country’s usage rate is modeled as a deviation from the overall mean of 85%, with partial pooling. About 95% of countries’ usage rates would fall between 63% and 97%.
$\sigma_{\phi \;}$	Between-group standard deviation of country-specific proportion of distributed HIVST kits used	~N(0, 0.5)	Weakly informative truncated normal distribution [0.000001, 5].

### Model calibration

The model was calibrated within a Bayesian framework with a hierarchical structure on rate ratio parameters, borrowing strength across countries, with weakly informative priors. The calibration outcomes include: *1)* the survey proportion of the population reporting having ever used an HIVST, stratified by sex and age groups (using a binomial likelihood), and *2)* the annual number of HIVST distributed (using a normal likelihood). For the binomial likelihood, we used design-adjusted effective sample sizes accounting for clusters, strata, and weights.

For each country, we calibrate the annual testing rate for females aged 15–24 years (i.e., the referent group), and the following time-invariant parameters (1) the male-to-female RR for the referent age group; (2) the male-specific RRs for each age group; (3) the female-specific RRs for each age group; (4) the proportion of distributed HIVST kits used; and (5) the HIVST re-testing RR. The sex- and age-related parameters are informed by the survey data (as the program data contains no information on sex or age of the users). An overlap between the survey and program data for a given year informs the proportion of HIVST used and the re-testing ratio; a single overlapping year informs the fraction of kits used, whereas multiple overlapping years are required to inform the re-testing rate. We also assumed that HIVST kits were used in the same calendar year they were distributed.

In addition, we incorporated a soft quadratic penalty in years where program data were missing to avoid unrealistically high HIVST testing rates. The penalty threshold was set for the model-predicted annual number of HIVST distributed to 20 times the size of 0.10% of the population for countries with maximum annual testing coverage below 0.10%, and to four times the maximum observed number of HIV tests for all other countries. We used these two thresholds because, in countries with very low testing volumes (i.e., <0.1% coverage per year), using four times the maximum number was too restrictive. In all cases, the thresholds were chosen not to impact the estimated parameters and only reduce implausible trajectories in years after the data point. To obtain posterior distributions for the estimated parameters, we used a Hamiltonian Monte Carlo (HMC) sampling algorithm with four chains and 4,000 iterations each (including 2,000 iterations for warm-up) implemented in the *rstan* package (version 2.32.7) [32]. We assessed convergence through traceplots and the R-hat statistics and summarized posterior distributions by the median and 95% credible intervals (95%CrI). Model performance was assessed using in-sample comparisons.

### Model outputs

Using the calibrated model, we calculated the proportion of the population that ever used an HIVST by country, year, sex, and age group, and the annual total number of HIVST kits used for each country from 2012 to 2024. To calculate the aggregated trends by age groups (14–24, 25–34, 35–49, ≥50 years), sex (men/women), and region (western and central Africa/eastern and southern Africa), the country and year-specific estimates for HIVST uptake were weighted by their relative population size.

All the analyses were performed using R software *(version 4.4.3)*. This study complies with the *Guidelines for Accurate and Transparent Health Estimates Reporting* (GATHER) statement (Table D in S1 Appendix).

### Ethics

All individuals participating in the survey provided verbal informed consent. The protocols for the DHS surveys have been approved by the Internal Review Board of ICF International in Calverton, USA. For all other surveys included in this study (MICS, PHIA, BAIS, and KAIS), approval was obtained from relevant country authorities. Ethical approval for secondary data analyses was obtained from the Institutional Review Board of McGill University, Canada (A10-E72-17B).

## Results

### Included population-based surveys and countries with HIVST program data

**Fig 2:**
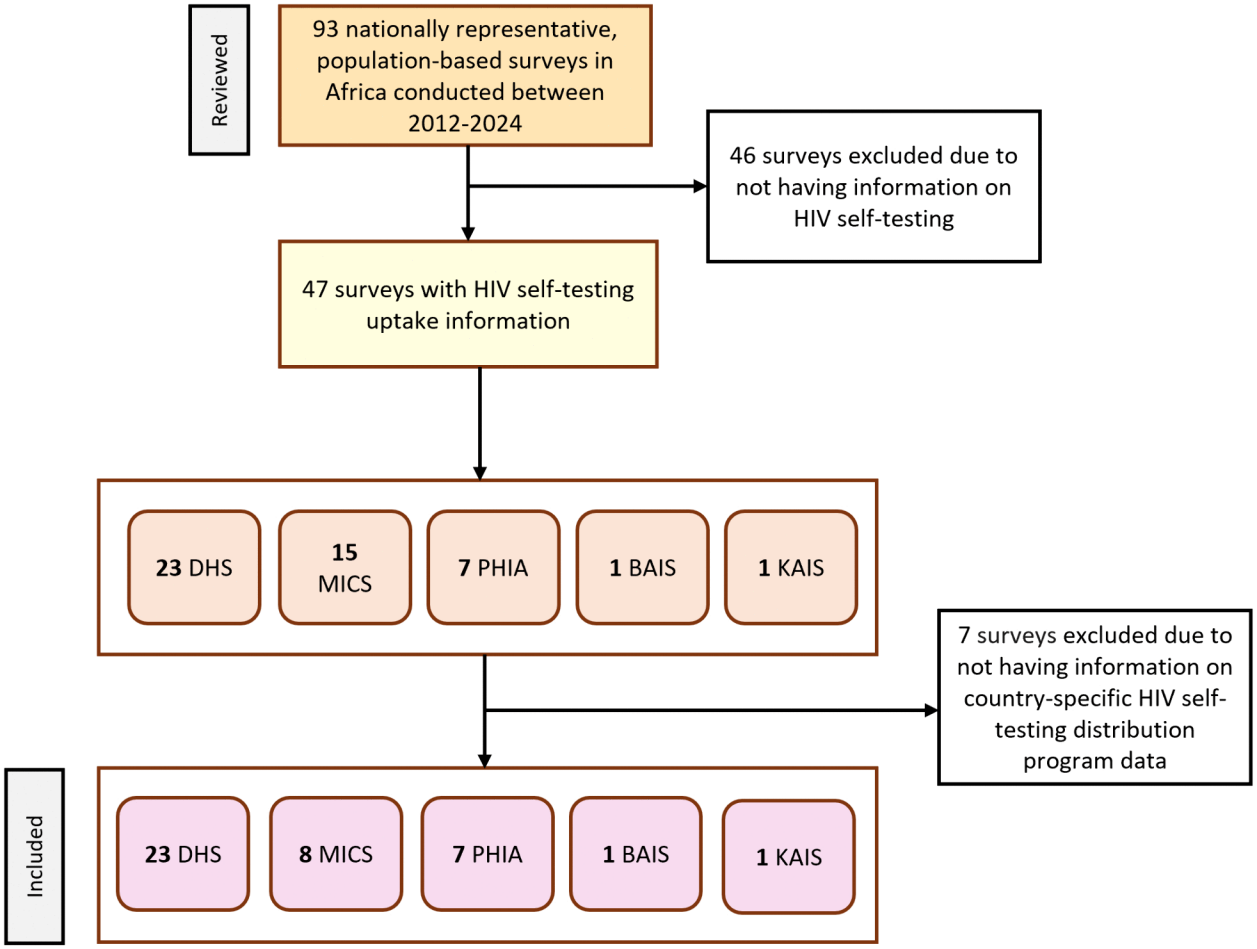
Flowchart of population-based surveys reviewed for inclusion to inform the model estimated proportion of HIV self-testing (HIVST) uptake in Africa (2012–2024). DHS: Demographic and Health Survey; MICS: Multiple Indicator Cluster Survey; PHIA: Population-based HIV Impact Survey; BAIS: Botswana AIDS Impact Survey; KAIS: Kenya AIDS Indicator Survey.

We reviewed 93 surveys in Africa from 2012 to 2024 and identified 47 surveys from 33 countries with self-reported HIVST usage. Of the surveys identified, there were 23 DHS, 15 MICS, 7 PHIA, 1 BAIS, and 1 KAIS surveys. Among the 33 countries with survey data, we excluded six that did not have any HIVST program data: the Central African Republic, Chad, Comoros, Gabon, The Gambia, Mauritania, and Togo. Among countries with program data, these were available for a median of 4 years, totaling 99 country-years of information on 20 million HIVST kits distributed. The final analysis included 40 surveys (23 DHS, 8 MICS, 7 PHIA, 1 KAIS, and 1 BAIS surveys) conducted in 27 countries containing information on 854,501 respondents aged ≥15 years, with 10 countries having more than one survey (Fig 2).

### Trends in HIVST uptake by region, sex, and age

**Fig 3:**
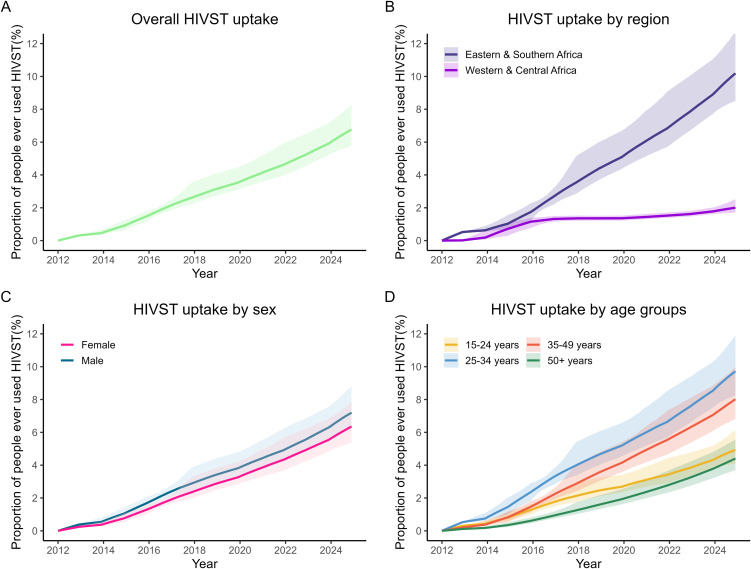
Model estimated trends in HIV self-testing (HIVST) uptake (i.e., proportion who ever used an HIVST) overall (A), by region (B), sex (C), and age groups (D) in Africa (2012–2024). The lines represent the median, and the shaded areas represent the 95%credible intervals.

Our model reliably reproduced HIVST usage estimates from population-based surveys and replicated annual HIVST distribution volumes (Figs B and C in S1 Appendix). In-sample comparisons yielded median errors near zero, confirming that the model is unbiased with appropriately centered credible intervals. However, these intervals were slightly wide for the program data and moderately narrow for the survey data (Table E in S1 Appendix). We estimated that, across the 27 African countries included, the proportion of adults aged ≥15 years who had ever tested for HIV using an HIVST (henceforth defined as HIVST uptake) increased steadily from <1% in 2012 to 6.8% (95%CrI [5.8, 8.2]) in 2024 (Fig 3A). Although the proportion of HIVST users increased overall, the progress differed by country and region. Before 2016, uptake was similar between regions, but thereafter the countries in western and central Africa had consistently lower uptake (2.0% in 2024; 95%CrI [1.7, 2.5]) than countries in eastern and southern Africa (10.2% in 2024; 95%CrI [8.5, 12.7]; Fig 3B).

HIVST uptake was slightly higher in men (7.2% in 2024; 95%CrI [6.1, 8.8]) than women (6.4% in 2024; 95%CrI [5.4, 7.8]; Fig 3C). Additionally, in 2024, HIVST uptake was estimated to be highest among 25–34-year-olds (9.7%; 95%CrI [8.2, 11.9]), followed by 35–49-year-olds (8.0%; 95CrI [6.8, 10]) and 15–24-year-olds (4.9%; 95%CrI [4.1, 6]). It was lowest among people aged ≥50 years (4.4%; 95%CrI [3.7, 5.6]; Fig 3D).

### Country-level estimates of HIVST uptake in 2024

**Fig 4:**
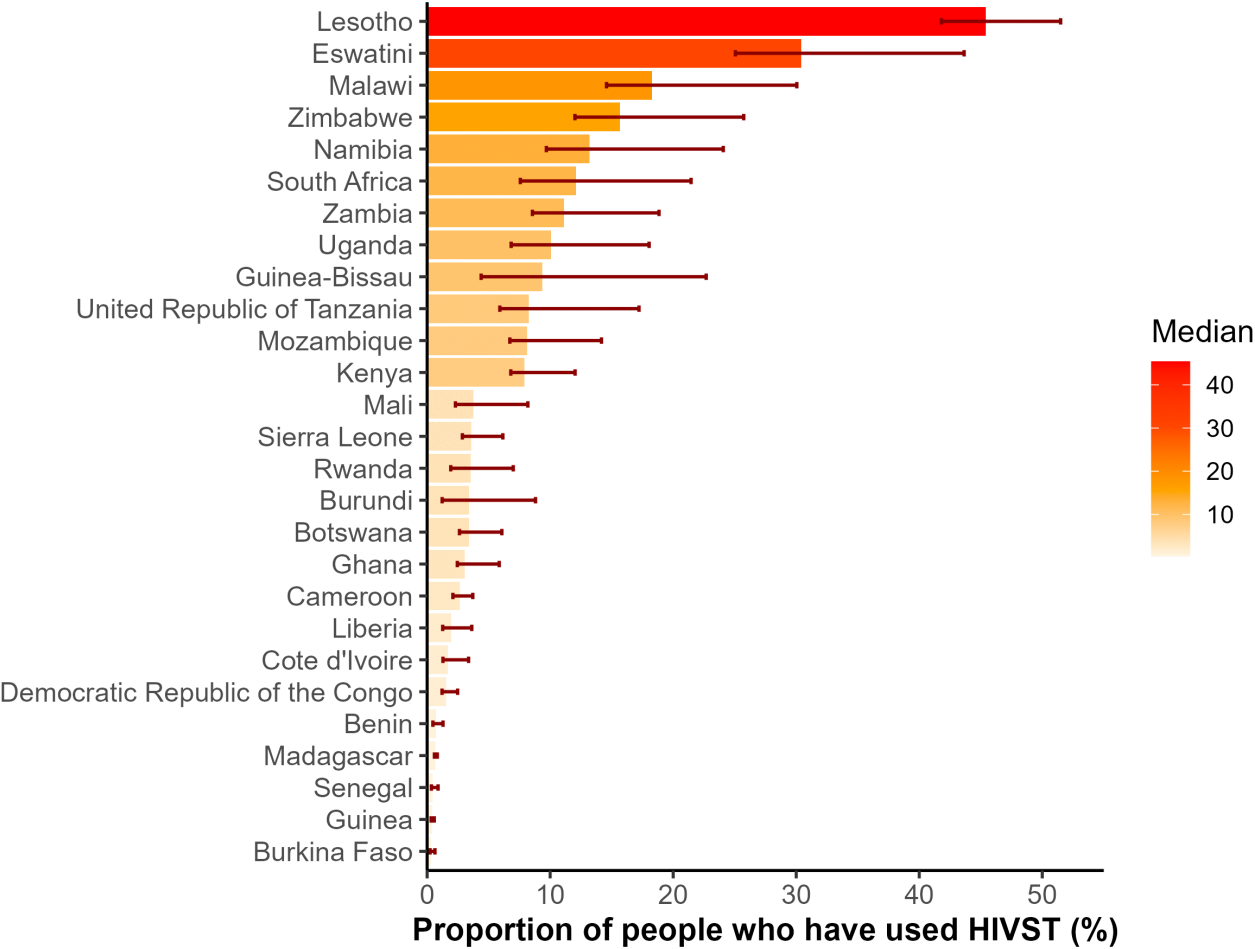
National estimates of HIV self-testing (HIVST) uptake (i.e., proportion of the population aged 15+ years who ever used an HIVST) in 2024. The medians represent the posterior median estimates of national HIVST uptake in 2024.

National estimates for the proportion of individuals reporting ever using an HIVST in 2024 were highly heterogeneous (Fig 4). In 2024, eastern and southern African countries had the highest national estimates of HIVST uptake (above 10%): Lesotho (45%; 95%CrI [42, 51]), Eswatini (30%; 95%CrI [25, 43]), Malawi (18%; 95%CrI [15, 30]), Zimbabwe (16%; 95%CrI [12, 26]), South Africa (12%; 95%CrI [8, 22]), Namibia (13%; 95%CrI [10, 24]), and Zambia (11%; 95%CrI [9, 19]) (Fig 4). In contrast, HIVST uptake as of 2024 was below 1% in the following countries: Madagascar, Benin, Guinea, Senegal, and Burkina Faso, which are countries with low national HIV prevalence (Fig 4).

### HIVST rate ratios by age and sex

**Fig 5:**
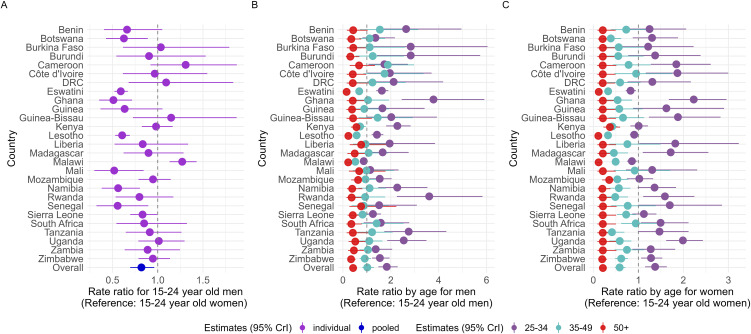
Overall and country-specific posterior distributions of the following parameters: rate ratios for 15–24-year-old men (A), age-specific rate ratios for men (B), age-specific rate ratios by age group for women (C). The filled circles represent the median estimates, and the horizontal bars represent the 95% credible intervals (CrI).

Among the youngest 15–24-year-olds age group, HIVST rate was lower in men (RR = 0.8; 95%CrI [0.7, 0.9]) than in women. However, there were substantial variations by country (Fig 5A). For both sexes, the pattern for the age-specific rate ratios was generally consistent across countries (Fig 5B, 5C), with 25–34-year-olds testing at higher rates than other age groups. In contrast, people aged ≥35 years had the lowest HIVST rates.

The pooled age-specific rate ratios for 25–34- and 35–49-year-olds differed between men and women. By age 25–34 years, men were 1.8 (95%CrI [1.5, 2.3]) times more likely to use HIVST than those 15–24-year-olds, while 25–34-years-old women were 1.4 (95%CrI [1.1, 1.6]) times more likely than 15–24-year-old women (Fig 5C). In the 35–49-year age group, the difference from the referent group for the overall age-specific rate ratios was less pronounced in men (RR = 1.0; 95%CrI [0.8, 1.3]; Fig 5B) than in women (RR = 0.6; 95%CrI [0.5, 0.7]; Fig 5C). Most population-based surveys only collected information on men and women aged 15–49 years, with some surveys including men aged 50+. As such, the rate ratios for that latter group were more precise than those of women.

### HIVST re-testing rate ratios and proportion of distributed HIVST kits used

**Fig 6:**
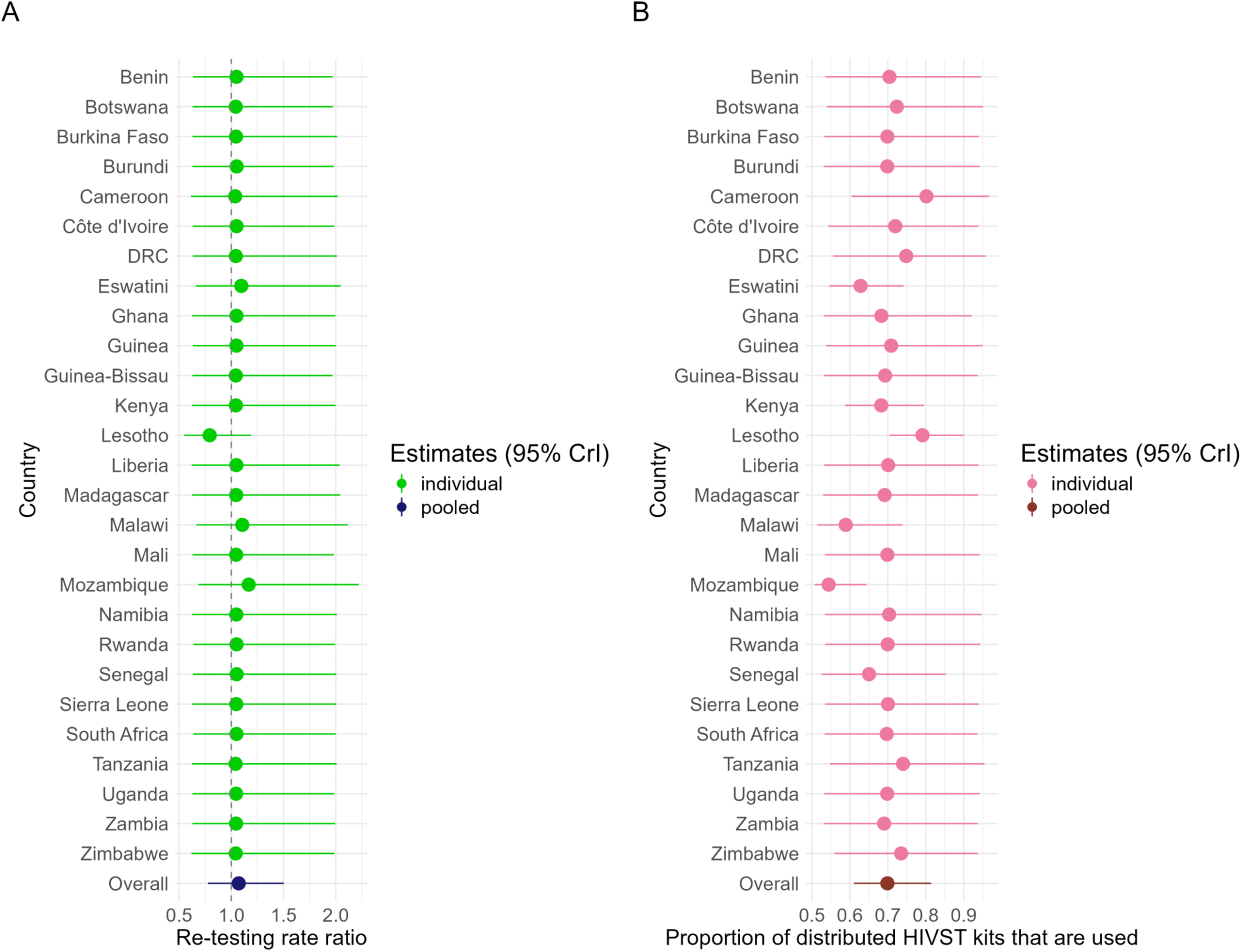
Overall and country-specific posterior distributions of the following parameters: re-testing rate ratio (A) and proportion of distributed kits used (B). The filled circles represent the median estimates, and the horizontal bars represent the 95% credible intervals (95%CrI).

Compared to first-time HIVST users, we estimated that people who had previously used an HIVST were slightly more likely (RR = 1.1; 95%CrI [0.8, 1.5]) to re-use one, with substantial uncertainty (Fig 6A). This parameter was imprecise because an accurate estimate requires a time series of survey and program data that overlap. This information was available from only four countries: Eswatini, Lesotho, Malawi, and Mozambique (Fig 6A).

The fraction of distributed kits that are used had slightly more information in the data, since it could be estimated when there was a minimum of 1 year of overlap in years of survey and years of HIV testing program data. Overall, we estimate that 70% (95%CrI [60, 80]) of distributed HIVST kits are used by end users (Fig 6B). Credible intervals were wide and overlapping for most countries (Fig 6B).

## Discussion

Tracking uptake of HIVST is challenging. Using 40 population-based surveys encompassing more than 850,000 participants and program data from 27 countries, we developed a flexible hierarchical mathematical model of HIVST behaviors. We estimated that as of 2024, 7% of people aged ≥15 years had ever used an HIVST in these countries. Our results suggest that males are more likely to use an HIVST, that individuals aged 25–34 have the highest rates of HIVST use, that about 70% of distributed kits are used, and that individuals who have used an HIVST in the past could be slightly more likely to use it again, though this specific estimate is not precise.

Across Africa, coverage of traditional facility-based HTS has consistently been lower for men compared to women, contributing to consistent gender disparities in HIV testing [36]. A modeling study estimated that men aged 35–49 years were the largest undiagnosed group of PLHIV unaware of their HIV serostatus in 2020 [1,37]. Our results indicate that men are slightly more likely to have used an HIVST (7.2% for men versus 6.4% for women). Such findings are consistent with existing literature [8,38]. Men’s higher uptake of HIVST could be the consequence of existing distribution strategies in Africa that prioritize them such as 1) distribution to partners of pregnant and breastfeeding women; 2) formal and informal workplace distributions; 3) prisons and harm reduction centers; 4) mobile brigades and outreach testing; 5) targeted distribution to key populations, especially men who have sex with men and men sex workers [3,39]. This points to the potential role that HIVST can play in closing HIV diagnosis gaps. Lower uptake of HIVST in younger age groups may also be related to policy barriers that limit access due to age of consent laws. While WHO has previously recommended these be reviewed and revised, several countries, particularly in west and central Africa, limit HIV testing and self-testing to those ≥18 years [40].

The scale-up of HIVST was highly variable across countries and regions. The eastern and southern regions, which have higher HIV prevalences, showed greater uptake of HIVST (10%)—five-times that of the western and central regions (2%). Countries where HIVST uptake exceeded 10% in 2024 were all part of the STAR initiative, except Namibia. National HIVST implementation follows two main strategies: 1) distribution prioritized to key populations (e.g., sex workers, men who have sex with men), and 2) facility-based distribution through public and private sectors. In practice, however, countries adopt a mix of distribution models [41]. Countries with higher HIV burden and more established ART programs have incorporated HIVST into long-term sustainability plans, using it to reach first-time testers and support routine re-testing among vulnerable populations. Irrespective of the strategy adopted, our results indicated that most of the distributed HIVST kits are used, although the pooled estimate has wide uncertainty. Our analysis triangulating survey and program distribution data assumed that HIVST kits were used in the same calendar year they were distributed. This may be inaccurate in situations where kits are intentionally retained for later use. Parameter identifiability was weaker in countries with sparse or nonoverlapping survey and program data, particularly for the re-testing rate ratio and the fraction of distributed kits used. In such settings, posterior estimates were more influenced by the hierarchical prior structure, which induces greater shrinkage toward the pooled effect and yields wider credible intervals. By contrast, parameters related to age and sex patterns were generally better identified because they were informed by multiple stratified survey observations.

Lesotho has the highest estimated HIVST uptake of all included countries in 2024. Self-testing in Lesotho has been widely implemented as a screening tool and as a substitute for provider-administered testing [42]. The HIVST delivery model in Lesotho employs both facility-based and community-level distribution models, but it primarily focuses on expanding testing access among the overall population, which likely resulted in our estimated re-testing rate ratio of less than one. On the other hand, in Eswatini, facility-based universal screening using HIVST is offered to males aged 20–34 years and females aged 15–24 years, along with peer distribution, community-led distribution, and social network distribution [43]. In this context, the high re-testing rate ratio estimated for Eswatini may be explained by the use of HIVST as a routine tool for specific priority populations and high-risk individuals who could be more likely to engage in frequent self-testing. For Mozambique, the higher re-testing rate ratio (>1.1) could reflect its key populations-focused HIVST implementation strategy. Within their strategy, there is limited pharmacy-based distribution, but a facility-based distribution of HIVST kits is currently being piloted with a large scale-up planned [44].

Most western and central African countries, such as Benin, Burkina Faso, Senegal, and the DRC, had lower HIVST uptake. In these countries, HIVST strategies have primarily focused on key populations. In Benin, Burkina Faso, and Senegal, HIVST was largely financed by the Global Fund, and distribution prioritized to men who have sex with men and female sex workers [45]. In the DRC, HIVST was funded by PEPFAR and includes some facility-based distribution—but the emphasis remained on key populations—and is also offered through index testing and partner-delivered strategies among sero-discordant couples [46,47]. The low uptake in these settings may be further threatened by the closure of *United States Agency for International Development* (USAID) supported drop-in centers for key populations because of changes to US foreign aid and PEPFAR priorities implemented in early 2025. Innovative uses of HIVST may be increasingly employed in settings with health worker shortages and limited budgets, as they offer cost savings and efficiencies; expanded access through pharmacies and client-led testing may also become more feasible and attractive under such constraints.

Our study results should be interpreted considering the following limitations. First, some of the model parameters are best informed in settings with a good temporal overlap between survey and program data. However, few countries met this criterion, which led to imprecise estimates of the HIVST re-testing rate ratio and, to a lesser extent, the proportion of distributed HIVST kits used. Second, survey data on HIVST use are self-reported and could be subject to recall bias and/or misclassification. For instance, individuals might misinterpret provider-initiated point-of-care rapid testing as self-testing. Third, we excluded six countries that did not have any HIVST program data. Countries that did not collect information on HIVST distribution could have lower HIVST usage, which would lead to overestimation of uptake in our aggregated estimates compared to the whole of the African region. Fourth, coverage of the credible intervals was wide for the HIVST program data and narrow for the survey data, albeit properly centered, reflecting differences in error structures. Finally, we did not model HIVST positivity and awareness of HIV status because most surveys did not collect HIV serostatus, and the HTS program data for most countries do not report confirmatory testing following a reactive HIVST.

Strengths of our study included assembling one of the most comprehensive datasets on HIVST uptake from population-based surveys and HIVST program data from African countries. Second, we calibrated the model to all 27 countries simultaneously in a Bayesian framework, improving estimation of important parameters in data sparse settings. Third, our model sheds light on HIVST behaviors through the synthesis and triangulation of two complementary data sources [48]. Our study provides evidence that HIVST is no longer a marginal HIV testing modality. Given the increasing use of HIVST, countries should continue measuring the uptake of this critical tool to understand gaps in coverage. Moving forward, epidemiological models that intend to estimate awareness of HIV status should incorporate this modality (at least in countries with moderate levels of HIVST use) for evaluating the progress towards achieving the ‘first 95’ target [1,30]. However, limited information exists on how HIVSTs are being implemented across countries and over time, which poses challenges to understand the impacts of this modality. Moreover, after the sudden dismantlement of major survey initiatives (such as DHS) in February 2025 by the United States, countries will increasingly rely on other multi-country survey initiatives (i.e., MICS) or smaller country-specific surveys that may be conducted less frequently, increasing our reliance on routinely collected program data on HIVST distribution [49]. Our modeling framework has the potential to strengthen the interpretability and usability of such program data.

Our findings can support countries in optimizing the use of HIVST by prioritizing individuals most likely to be undiagnosed, thus sustaining HTS amid shifting data landscapes and funding priorities. Our modeling framework offers a practical tool for national HIV programs to monitor HIVST uptake, and guide policy decisions and interventions aimed at increasing HIV testing coverage and improving linkage to care to, ultimately, reduce HIV incidence.

## Supporting information

S1 Appendix Supplementary materials presenting additional survey information, model equations, calibration and model fits, and sensitivity analyses. (PDF)

## Abbreviations

95%CrI: 95% credible interval
AIS: AIDS Indicator Cluster Surveys
BAIS: Botswana AIDS Impact Survey
DHS: Demographic and Health Surveys
GAM: Global AIDS Monitoring
GATHER: Guidelines for Accurate and Transparent Health Estimates Reporting
HIVST: HIV self-testing
HMC: Hamiltonian Monte Carlo
HTS: HIV testing services
KAIS: Kenya AIDS Indicator Survey
MICS: Multiple Indicator Cluster Surveys
PHIA: Population-based HIV Impact Assessments
PLHIV: people living with HIV
RR: rate ratio
USAID: United States Agency for International Development
WHO: World Health Organization
WPP: World Population Prospects.
