# Supplementary material for: Trends in HIV self-testing uptake in Africa: A modeling study of population-based surveys and HIV testing program data
Source: PLoS Med. 2026 May 5;23(5):e1004771. doi: 10.1371/journal.pmed.1004771 (PMC13143066; doi:10.1371/journal.pmed.1004771)
Supplement: S1 Appendix — (PDF) [file pmed.1004771.s001.pdf]

Supplementary Materials

Trends in HIV self-testing uptake in Africa: a modeling study of population-based surveys and HIV testing program data

Table A. List of survey questions included in the population-based surveys.

Country	Survey	Year	Survey Item/Question	Respondents
**Benin**	DHS	2017	Vous êtes-vous testée vous-même en utilisant un kit de test?	• Women (15-49 years)<br>• Men (15-64 years)
**Botswana**	BAIS	2021	Have you ever tested yourself for HIV using a self-test kit?	• Women (15-50+ years)<br>• Men (15-50+ years)
**Burkina Faso**	DHS	2021	Vous êtes-vous testée vous-même pour le VIH en utilisant un kit d'autotest?	• Women (15-49 years)<br>• Men (15-59 years)
**Burundi**	DHS	2016	Vous êtes-vous testée vous-même en utilisant un kit de test?	• Women (15-49 years)<br>• Men (15-59 years)
**Cameroon**	DHS	2018	Vous êtes-vous testée vous-même en utilisant un kit de test?	• Women (15-49 years)<br>• Men (15-64 years)
**Cote d'Ivoire**	DHS	2021	Vous êtes-vous testée vous-même en utilisant un kit de test?	• Women (15-49 years)<br>• Men (15-59 years)
**Democratic Republic of the Congo (DRC)**	MICS	2018	Est-ce que vous vous êtes déjà testée vous-même pour le VIH en utilisant ces kits?	• Women (15-49 years)<br>• Men (15-49 years)
**Eswatini**	PHIA	2021	Have you ever tested yourself for HIV using s self-test kit?	• Women (15-50+ years)<br>• Men (15-50+ years)
**Eswatini**	MICS	2022	Have you ever tested yourself for HIV using a self-test kit?	• Women (15-49 years)<br>• Men (15-49 years)
**Ghana**	MICS	2017	Have you ever tested yourself for HIV using a self-test kit?	• Women (15-49 years)<br>• Men (15-49 years)
**Ghana**	DHS	2022	Have you ever tested yourself for HIV using a self-test kit?	• Women (15-49 years)<br>• Men (15-59 years)
**Guinea**	DHS	2018	Vous êtes-vous testée vous-même en utilisant un kit de test?	• Women (15-49 years)<br>• Men (15-59 years)
**Guinea-Bissau**	MICS	2018	Será que já fizeste pessoalmente o teste de VIH utilizando estes kits?	• Women (15-49 years)<br>• Men (15-49 years)
**Kenya**	KAIS	2012	Have you ever tested yourself for HIV in private using a self-test kit?	• Women (15-64 years)<br>• Men (15-64 years)
**Kenya**	PHIA	2018	Have you ever tested yourself for HIV in private using a self-test kit?	• Women (15-50+ years)<br>• Men (15-50+ years)
**Kenya**	DHS	2022	Have you ever tested yourself for HIV using a self-test kit?	• Women (15-49 years)<br>• Men (15-54 years)
**Lesotho**	PHIA	2020	Have you ever tested yourself for HIV using s self-test kit?	• Women (15-50+ years)<br>• Men (15-50+ years)
**Lesotho**	DHS	2023	Have you ever tested yourself for HIV using a self-test kit?	• Women (15-49 years)<br>• Men (15-59 years)
**Liberia**	DHS	2019	Have you ever tested yourself for HIV using a self-test kit?	• Women (15-49 years)<br>• Men (15-59 years)

**Madagascar**	MICS	2018	Est-ce que vous vous êtes déjà testée vous-même pour le VIH en utilisant ces kits?	• Women (15-49 years)<br>• Men (15-49 years)
**Madagascar**	DHS	2021	Vous êtes-vous testée vous-même en utilisant un kit de test?	• Women (15-49 years)<br>• Men (15-59 years)
**Malawi**	DHS	2015	Have you ever tested yourself for HIV using a self-test kit?	• Women (15-49 years)<br>• Men (15-54 years)
**Malawi**	MICS	2019	Have you ever tested yourself for HIV using a self-test kit?	• Women (15-49 years)<br>• Men (15-49 years)
**Malawi**	PHIA	2020	Have you ever tested yourself for HIV using s self-test kit?	• Women (15-50+ years)<br>• Men (15-50+ years)
**Mali**	DHS	2018	Vous êtes-vous testée vous-même en utilisant un kit de test?	• Women (15-49 years)<br>• Men (15-59 years)
**Mozambique**	PHIA	2021	Have you ever tested yourself for HIV using s self-test kit?	• Women (15-50+ years)<br>• Men (15-50+ years)
**Mozambique**	DHS	2022	Alguma vez na vida fez o teste de HIV, usando um kit de autoteste?	• Women (15-49 years)<br>• Men (15-54 years)
**Namibia**	PHIA	2017	Have you ever tested yourself for HIV in private using a self-test kit?	• Women (15-64 years)<br>• Men (15-64 years)
**Rwanda**	DHS	2019	Have you ever tested yourself for HIV using a self-test kit?	• Women (15-49 years)<br>• Men (15-59 years)
**Senegal**	DHS	2017	Vous êtes-vous testée vous-même en utilisant un kit de test?	• Women (15-49 years)<br>• Men (15-59 years)
**Senegal**	DHS	2023	Vous êtes-vous testée vous-même en utilisant un kit de test ?	• Women (15-49 years)<br>• Men (15-59 years)
**Sierra Leone**	MICS	2017	Have you ever tested yourself for HIV using a self-test kit?	• Women (15-49 years)<br>• Men (15-49 years)
**Sierra Leone**	DHS	2019	Have you ever tested yourself for HIV using a self-test kit?	• Women (15-49 years)<br>• Men (15-59 years)
**South Africa**	DHS	2016	Have you ever tested yourself for HIV using a self-test kit?	• Women (15-49 years)<br>• Men (15-59 years)
**Tanzania**	DHS	2022	Have you ever tested yourself for HIV using a self-test kit?	• Women (15-49 years)<br>• Men (15-49 years)
**Uganda**	DHS	2016	Have you ever tested yourself for HIV using a self-test kit?	• Women (15-49 years)<br>• Men (15-54 years)
**Zambia**	DHS	2018	Have you ever tested yourself for HIV using a self-test kit?	• Women (15-49 years)<br>• Men (15-59 years)
**Zimbabwe**	DHS	2015	Have you ever tested yourself for HIV using a self-test kit?	• Women (15-49 years)<br>• Men (15-54 years)
**Zimbabwe**	MICS	2019	Have you ever tested yourself for HIV using a self-test kit?	• Women (15-49 years)<br>• Men (15-49 years)
**Zimbabwe**	PHIA	2020	Have you ever tested yourself for HIV using s self-test kit?	• Women (15-50+ years)<br>• Men (15-50+ years)

**BAIS = Botswana AIDS Indicator Survey, DHS = Demographic and Health Survey, KAIS = Kenya AIDS Indicator Survey, MICS = Multiple Indicator Cluster Survey, PHIA = Population-based HIV Impact Assessment*

The individual-level data from the population-based surveys included in our analyses are available from the following sources upon request:

- Botswana AIDS Indicator Survey 2021 (https://statsbots.org.bw/).
- Demographic and Health Surveys (https://dhsprogram.com/Data/).
- Kenya AIDS Indicator Survey 2012 (https://statistics.knbs.or.ke/nada).

- Multiple Indicator Cluster Surveys (https://mics.unicef.org/surveys).
- Population-based HIV Impact Assessment (https://phia-data.icap.columbia.edu/datasets).

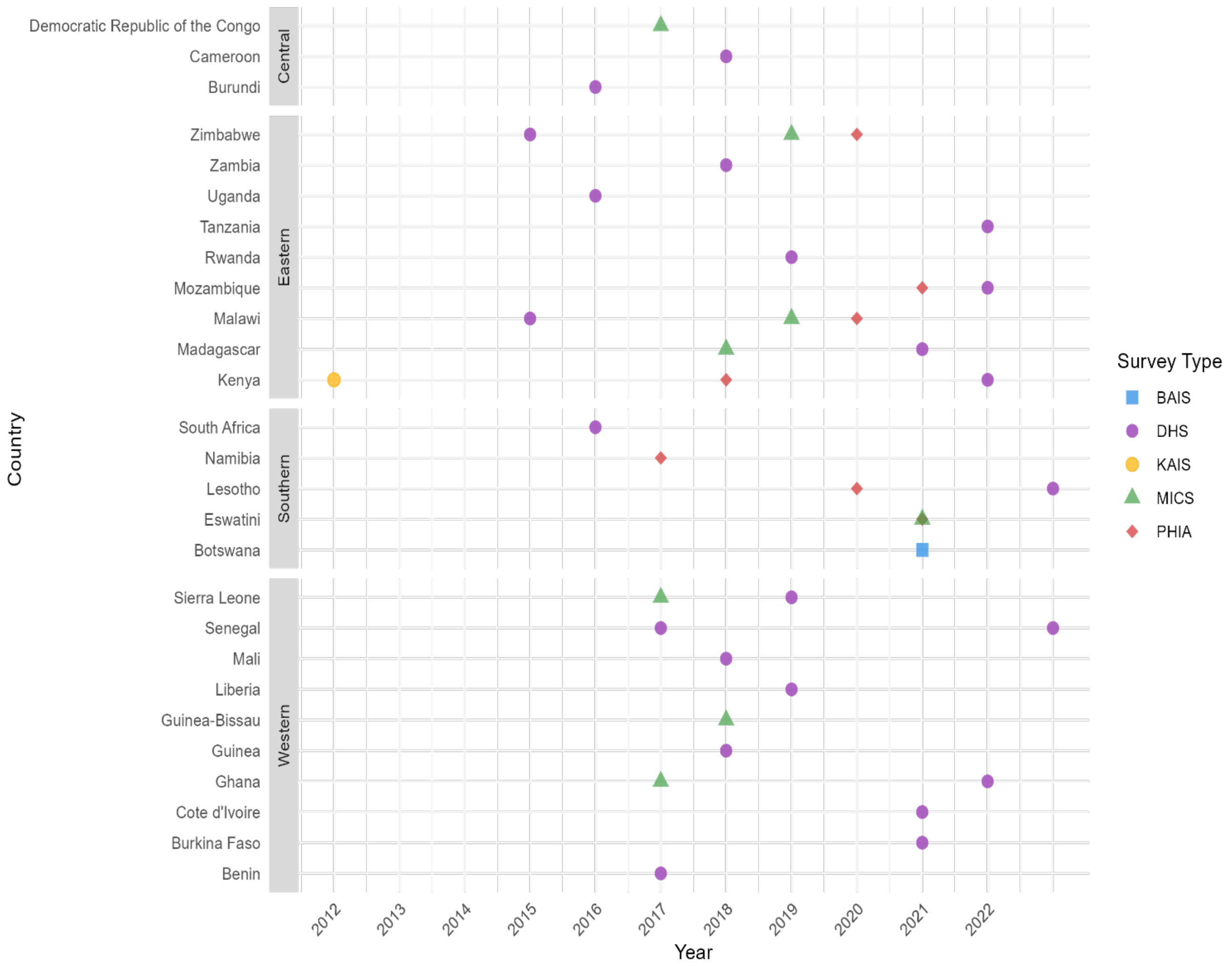

Fig A. Data availability for proportion of people who have ever used an HIVST by country and year (grouped by region) in Africa, 2012 - 2024. DHS: Demographic and Health Survey; MICS: Multiple Indicator Cluster Survey; PHIA: Population-based HIV Impact Survey; BAIS: Botswana AIDS Impact Survey; KAIS: Kenya AIDS Indicator Survey.

Table B. Survey-specific HIVST awareness and uptake, with 95% confidence intervals of the surveys included.

Country	Year	Survey	Sample Size	Knowledge of HIVST (%) with 95% CI	Ever used an HIVST (%) with 95% CI
**Benin**	2017	DHS	23,523	4.5 (4.1-4.9)	0.6 (0.5-0.7)
**Botswana**	2021	BAIS	17,205	-	2.1 (1.8 - 2.5)
**Burkina Faso**	2021	DHS	25,379	3.9 (3.6 - 4.4)	0.3 (0.2 - 0.4)
**Burundi**	2016	DHS	24,821	3.9 (3.5 - 4.5)	0.3 (0.2 - 0.4)
**Cameroon**	2018	DHS	21,655	15.8 (14.7 - 17.0)	2.5 (2.2 - 2.9)
**Cote d'Ivoire**	2021	DHS	22,468	10.2 (9.1 - 11.3)	1.1 (0.9 - 1.4)
**Democratic Republic of the Congo (DRC)**	2017	MICS	27,858	11.2 (9.9 - 12.8)	2.0 (1.6 - 2.6)
**Eswatini**	2021	PHIA	12,043	-	20.0 (18.8 - 21.3)
**Eswatini**	2021	MICS	3,659	71.9 (70.0 - 73.7)	28.6 (26.6 - 30.5)
**Ghana**	2017	MICS	19,693	15.8 (14.9 - 16.8)	2.1 (1.8 - 2.5)
**Ghana**	2022	DHS	22,058	19.7 (18.8 - 20.7)	2.2 (1.9 - 2.5)
**Guinea**	2018	DHS	14,991	7.3 (6.6 - 8.1)	0.7 (0.5 -1.0)
**Guinea-Bissau**	2018	MICS	13,750	7.9 (7.0 - 8.8)	2.8 (2.2 - 3.5)
**Kenya**	2022	DHS	46,609	39.9 (39.2 - 40.7)	6.17 (5.8 - 6.56)
**Kenya**	2018	PHIA	42,452	-	4.1 (3.7 - 4.6)
**Kenya**	2012	KAIS	13,694	-	3.4 (3.0 – 4.0)
**Lesotho**	2020	PHIA	16,468	-	9.3 (8.7 - 10.0)
**Lesotho**	2023	DHS	1,856	60.7 (57.4 - 63.3)	34.9 (33.0 - 35.1)
**Liberia**	2019	DHS	12,314	11.0 (9.9 - 12.3)	1.4 (1.1 - 1.8)
**Madagascar**	2018	MICS	24,782	5.5 (4.9 - 6.1)	1.5 (1.2 - 1.9)
**Madagascar**	2021	DHS	27,906	3.4 (3.1 - 3.7)	0.4 (0.3 - 0.6)
**Malawi**	2015	DHS	32,040	10.4 (9.6 - 11.2)	1.0 (0.8 - 1.1)
**Malawi**	2019	MICS	31,328	36.7 (35.5 - 37.9)	7.4 (6.8 - 7.9)
**Malawi**	2020	PHIA	26,519	31.7 (30.4 - 32.9)	22.3 (20.9 - 23.7)
**Mali**	2018	DHS	15,137	7.4 (6.6 - 8.3)	0.8 (0.6 - 1.2)
**Mozambique**	2021	PHIA	17,105	-	9.9 (8.7 - 11.2)
**Mozambique**	2022	DHS	18,563	12.0 (11.1-12.9)	2.5 (2.2-2.8)
**Namibia**	2017	PHIA	26,521	-	4.1 (3.5 - 4.7)
**Rwanda**	2019	DHS	21,147	19.1 (18.3 - 19.9)	1.4 (1.2 - 1.6)
**Senegal**	2017	DHS	23,764	5.1 (4.6 - 5.7)	0.2 (0.1 - 0.3)
**Senegal**	2023	DHS	22,904	6.1 (5.5-6.9)	0.4 (0.3-0.6)
**Sierra Leone**	2017	MICS	25,259	16.4 (15.4 - 17.6)	2.9 (2.5 - 3.3)
**Sierra Leone**	2019	DHS	22,771	19.7 (18.3 - 21.3)	3.3 (2.8 - 3.9)

**South Africa**	2016	DHS	12,132	24.2 (22.8 - 25.7)	2.9 (2.5 - 3.4)
**Tanzania**	2022	DHS	21,017	21.3 (20.0 - 22.6)	3.6 (3.2 - 4.0)
**Uganda**	2016	DHS	23,842	25.7 (24.7 - 26.8)	4.8 (4.4 - 5.3)
**Zambia**	2018	DHS	25,815	20.4 (19.0 - 21.9)	2.8 (2.3 - 3.4)
**Zimbabwe**	2015	DHS	18,351	9.2 (8.6 - 9.8)	0.89 (0.7 - 1.1)
**Zimbabwe**	2019	MICS	14,309	32.1 (30.6 - 33.5)	5.5 (4.8 - 6.2)
**Zimbabwe**	2020	PHIA	20,793	-	6.1 (5.5 - 6.8)

Table C. Response rates of all included population-based surveys.

Country	Survey	Year	Response Rates of the Respondents (%)
**Benin**	DHS	2017	• Women (15-49 years): 97.2<br>• Men (15-64 years): 96.6
**Botswana**	BAIS	2021	• Women (15-50+ years): 86.8<br>• Men (15-50+ years): 78.3
**Burkina Faso**	DHS	2021	• Women (15-49 years): 97.9<br>• Men (15-59 years): 97.4
**Burundi**	DHS	2016	• Women (15-49 years): 98.5<br>• Men (15-59 years): 97.2
**Cameroon**	DHS	2018	• Women (15-49 years): 97.5<br>• Men (15-64 years): 96.9
**Cote d'Ivoire**	DHS	2021	• Women (15-49 years): 97.4<br>• Men (15-59 years): 96.7
**Democratic Republic of the Congo (DRC)**	MICS	2018	• Women (15-49 years): 99.7<br>• Men (15-49 years): 99.2
**Eswatini**	PHIA	2021	• Women (15-50+ years): 93.7<br>• Men (15-50+ years): 86.7
**Eswatini**	MICS	2022	• Women (15-49 years): 94<br>• Men (15-49 years): 83
**Ghana**	MICS	2017	• Women (15-49 years): 98.4<br>• Men (15-49 years): 97.2
**Ghana**	DHS	2022	• Women (15-49 years): 97.3<br>• Men (15-59 years): 96.2
**Guinea**	DHS	2018	• Women (15-49 years): 98.1<br>• Men (15-59 years): 96.2
**Guinea-Bissau**	MICS	2018	• Women (15-49 years): 97.8<br>• Men (15-49 years): 92.6
**Kenya**	KAIS	2012	• Women (15-64 years): 89.1<br>• Men (15-64 years): 77.4
**Kenya**	PHIA	2018	• Women (15-50+ years): 94.3<br>• Men (15-50+ years): 86.1
**Kenya**	DHS	2022	• Women (15-49 years): 92.9<br>• Men (15-54 years): 85.4
**Lesotho**	PHIA	2020	• Women (15-50+ years): 96.1<br>• Men (15-50+ years): 89.3
**Lesotho**	DHS	2023	• Women (15-49 years): 97.7<br>• Men (15-59 years): 96.9
**Liberia**	DHS	2019	• Women (15-49 years): 95.0<br>• Men (15-59 years): 92.3
**Madagascar**	MICS	2018	• Women (15-49 years): 91.0<br>• Men (15-49 years): 85.0
**Madagascar**	DHS	2021	• Women (15-49 years): 93.6<br>• Men (15-59 years): 89.8
**Malawi**	DHS	2015	• Women (15-49 years): 95.4<br>• Men (15-54 years): 88.1

**Malawi**	MICS	2019	• Women (15-49 years): 96.0<br>• Men (15-49 years): 88.0
**Malawi**	PHIA	2020	• Women (15-50+ years): 91.7<br>• Men (15-50+ years): 83.5
**Mali**	DHS	2018	• Women (15-49 years): 97.2<br>• Men (15-59 years): 95.7
**Mozambique**	PHIA	2021	• Women (15-50+ years): 88.7<br>• Men (15-50+ years): 84.3
**Mozambique**	DHS	2022	• Women (15-49 years): 91.8<br>• Men (15-54 years): 83.2
**Namibia**	PHIA	2017	• Women (15-64 years): 89.8<br>• Men (15-64 years): 81.2
**Rwanda**	DHS	2019	• Women (15-49 years): 99.7<br>• Men (15-59 years): 99.5
**Senegal**	DHS	2017	• Women (15-49 years): 93.9<br>• Men (15-59 years): 88.8
**Senegal**	DHS	2023	• Women (15-49 years): 93.1<br>• Men (15-59 years): 88.7
**Sierra Leone**	MICS	2017	• Women (15-49 years): 99.3<br>• Men (15-49 years): 98.4
**Sierra Leone**	DHS	2019	• Women (15-49 years): 95.3<br>• Men (15-59 years): 95.6
**South Africa**	DHS	2016	• Women (15-49 years): 71.9<br>• Men (15-59 years): 60.7
**Tanzania**	DHS	2022	• Women (15-49 years): 95.9<br>• Men (15-49 years): 89.3
**Uganda**	DHS	2016	• Women (15-49 years): 95.2<br>• Men (15-54 years): 92.3
**Zambia**	DHS	2018	• Women (15-49 years): 95.6<br>• Men (15-59 years): 90.8
**Zimbabwe**	DHS	2015	• Women (15-49 years): 95.1<br>• Men (15-54 years): 90.9
**Zimbabwe**	MICS	2019	• Women (15-49 years): 94.6<br>• Men (15-49 years): 89.3
**Zimbabwe**	PHIA	2020	• Women (15-50+ years): 94.5<br>• Men (15-50+ years): 86.5

Table D. Checklist of items for the Guidelines for Accurate and Transparent Health Estimates Reporting (GATHER).

Item #	Checklist item	Reported in section
**Objectives and funding**		
**1**	Define the indicator(s), populations (including age, sex, and geographic entities), and time period(s) for which estimates were made.	Tables S1 and S2
**2**	List the funding sources for the work.	Funding
**Data Inputs**		
For all data inputs from multiple sources that are synthesized as part of the study:		
**3**	Describe how the data were identified and how the data were accessed.	Methods: Data sources
**4**	Specify the inclusion and exclusion criteria. Identify all ad-hoc exclusions.	Methods: Data sources
**5**	Provide information on all included data sources and their main characteristics. For each data source used, report reference information or contact name/institution, population represented, data collection method, year(s) of data collection, sex and age range, diagnostic criteria or measurement method, and sample size, as relevant.	Table S1
**6**	Identify and describe any categories of input data that have potentially important biases (e.g., based on characteristics listed in item 5).	N/A
For data inputs that contribute to the analysis but were not synthesized as part of the study:		
**7**	Describe and give sources for any other data inputs.	Methods
For all data inputs:		
**8**	Provide all data inputs in a file format from which data can be efficiently extracted (e.g., a spreadsheet rather than a PDF), including all relevant meta-data listed in item 5. For any data inputs that cannot be shared because of ethical or legal reasons, such as third-party ownership, provide a contact name or the name of the institution that retains the right to the data.	GitHub repository
**Data analysis**		
**9**	Provide a conceptual overview of the data analysis method. A diagram may be helpful.	Methods
**10**	Provide a detailed description of all steps of the analysis, including mathematical formulae. This description should cover, as relevant, data cleaning, data pre-processing, data adjustments and weighting of data sources, and mathematical or statistical model(s).	Methods + Supplementary materials
**11**	Describe how candidate models were evaluated and how the final model(s) were selected.	N/A
**12**	Provide the results of an evaluation of model performance, if done, as well as the results of any relevant sensitivity analysis.	Supplementary materials: Figures S2 and S3, Table S5.
**13**	Describe methods for calculating uncertainty of the estimates. State which sources of uncertainty were, and were not, accounted for in the uncertainty analysis.	Methods + Supplementary materials
**14**	State how analytic or statistical source code used to generate estimates can be accessed.	GitHub repository
**Results and Discussion**		
**15**	Provide published estimates in a file format from which data can be efficiently extracted.	GitHub repository

Item #	Checklist item	Reported in section
**16**	Report a quantitative measure of the uncertainty of the estimates (e.g. uncertainty intervals).	Results
**17**	Interpret results in light of existing evidence. If updating a previous set of estimates, describe the reasons for changes in estimates.	Discussion
**18**	Discuss limitations of the estimates. Include a discussion of any modelling assumptions or data limitations that affect interpretation of the estimates.	Discussion

Text A. Description of the mathematical model for estimating regional and national trends of HIVST coverage.

We developed a mathematical model of HIVST behaviors and calibrated it to survey and program data using a Bayesian framework to estimate the model's parameters.

Model equations

The model uses a set of differential equations to simulate the proportion of the population who has ever used an HIVST over time by age and sex, and the number of annual HIVSTs distributed per year. The ordinary differential equations track changes in the number of individuals who have never used an HIVST ($N_{c,j,a}$) and those who have ever used an HIVST ($H_{c,j,a}$) for country *c*, sex *j (j=1 for females, j=2 for males)* and age group *a* (where *a=1* represents 15–24 years, *a=2* represents 25–34 years*, a=3* represents 35–49 years, and *a=4* represents above 50 years).

$$\frac{dN_{c,j,a=1}}{dt} = \varepsilon_{c,j}(t)\left(N_{c,j,a} + H_{c,j,a}\right) - \lambda_{c,j,a}(t)N_{c,j,a} - \propto_a N_{c,j,a} - \delta_{c,j,a}(t)N_{c,j,a}$$

$$\frac{dN_{c,j,a=\in\{2,3\}}}{dt} = \propto_{a-1} N_{c,j,a-1} - \lambda_{c,j,a}(t)N_{c,j,a} - \delta_{c,j,a}(t)N_{c,j,a} - \propto_a N_{c,j,a}$$

$$\frac{dN_{c,j,a=4}}{dt} = \propto_{a-1} N_{c,j,a-1} - \lambda_{c,j,a}(t)N_{c,j,a} - \delta_{c,j,a}(t)N_{c,j,a}$$

$$\frac{dH_{c,j,a=1}}{dt} = \lambda_{c,j,a}(t)N_{c,j,a} - \propto_a (t)H_{c,j,a} - \delta_{c,j,a}(t)H_{c,j,a}$$

$$\frac{dH_{c,j,a=\in\{2,3\}}}{dt} = \propto_{a-1} N_{c,j,a-1} + \lambda_{c,j,a}(t)N_{c,j,a} - \propto_a H_{c,j,a} - \delta_{c,j,a}(t)\, H_{c,j,a}$$

$$\frac{dH_{c,j,a=4}}{dt} = \propto_{a-1} N_{c,j,a-1} + \lambda_{c,j,a}(t)N_{c,j,a} - \delta_{c,j,a}(t)H_{c,j,a}$$

where the HIVST rate ($\lambda_{c,j,a}(t)$) is defined based on:

$$\lambda_{c,j,a}(t) = \begin{cases} \tau_{c,j}(t) \times RR_{c,a}^{age}; & if\ j = female \\ \tau_{c,j}(t) \times RR_{c,a}^{age} \times RR_{c,j}^{m-to-f}; & if\ j = male \end{cases}$$

And where the parameters in the system of ordinary differential equations are defined as:

$\varepsilon_{c,j}(t)$: yearly entry rate of 15-year-olds for country *c* and sex *j* at time *t*;

$\propto_a$: aging rate for age group *a* (fixed across countries, sexes and time); $\propto_a = \begin{cases} \frac{1}{10}; & if\ a = 1,2 \\ \frac{1}{15}; & if\ a = 3 \end{cases}$ $\delta_{c,j,a}(t)$: annual death rate for country *c*, sex *j* and age group *a* at time *t*;

$\tau_{c,j}(t)$: annual HIV self-testing rate for country *c*, sex *j* and at time *t*;

$RR_{c,j}^{sex}$: HIV self-testing rate ratio for males of age group 15-24 (referent is female aged 15-24 years);

$$RR_{c,j}^{sex} = \begin{cases} 1; & if\ j = 1 \\ calibrated; & if\ j = 2 \end{cases}$$

$RR_{c,a}^{age}$: HIV self-testing rate ratio for age groups (referent is 15-24 year old age group).

$$RR_{c,a}^{age} = \begin{cases} 1; & if\ a = 1 \\ calibrated; & if\ a = 2 \\ calibrated; & if\ a = 3 \\ calibrated; & if\ a = 4 \end{cases}$$

Priors

We modeled the *country-specific (c) testing rate* parameter ($\tau_c(t)$) for females aged 15-24 (i.e., the referent group) as a first-order random walk (RW) process.

$$log(\tau_c(1)) \sim N(-10,\ 1)$$

$$log(\tau_c(t > 1)) \sim N(log(\tau_c(t-1)),\ \sigma_{RW})$$

$$\sigma_{RW} \sim N(0, 0.25)\ \ with\ \ \sigma_{RW} > 0$$

Country-specific male-to-female testing rate ratios of the 15-24-year-olds were modeled as a combination of an average rate ratio $RR_{overall}^{m-to-f}$ and country-specific random effects RRn_c^{m-to-f} to pool information across countries. To improve convergence, we adopted a non-centered hierarchical parameterization, where the overall prior for the male-to-female rate ratio is 1 with 95% of the priors' density between rate ratios of 0.38 and 2.66.

$$\log(RR_{overall}^{m-to-f}) \sim N(0, 0.5)$$

$$RRn_c^{m-to-f} \sim N(0,1)$$

$$\sigma_{RR_{m-to-f}} \sim N(0, 0.5)\ \ with\ \ \sigma_{RR_{m-to-f}} > 0$$

$$log(RR_{c,j}^{male}) = \log(RR_{overall}^{m-to-f}) + \sigma_{RR_{m-to-f}}\ RRn_c^{m-to-f}$$

$RR_{overall}^{m-to-f}$: is the overall 15–24-year-old male-to-female HIVST rate ratio;

RRn_c^{m-to-f}: is the standard normal variable for country *c*;

$\sigma_{RR_{male}}$: is the scale parameter for RR_c^{male}, the between-group standard deviation;

$RR_{c,j}^{male}$: is the country-specific male-to-female HIVST rate ratio.

The model allowed for HIVST uptake to vary by age. To allow the age-specific rate ratios to differ by sex, country-specific age rate ratios were modeled hierarchically for males and females separately. Here again, a non-centered parameterization was used where the overall prior for the age rate ratios is 1, with 95% of the priors' density between rate ratios of 0.38 and 2.66.

$$log(RRoverall_{a,j}^{age}) \sim N(0, 0.5)$$

$$RRn_{c,a,j}^{age} \sim N(0,1)$$

$$\sigma_{RR_a^{age}} \sim N(0, 0.5)\ \ with\ \ \sigma_{RR_a^{age}} > 0$$

$$\log(RR^{age}_{c,a,j}) = \log(RRoverall^{age}_{a,j}) + \sigma_{RR^{age}_a} RRn^{age}_{c,a,j}$$

$RRoverall^{age}_{a,j}$: is the overall age-specific HIVST rate ratio for age group *a* and sex *j*;

$RRn^{age}_{c,a,j}$: is the standard normal variable for country *c,* age group *a*, and sex *j*;

$\sigma_{RR^{age}_a}$: is the scale parameter for $RRn^{age}_{c,a,j}$, the between-group standard deviation;

$RR^{age}_{c,a,j}$: contains the country-specific rate ratios by age and sex.

If HIVSTs are distributed to the same group of individuals, such as members of key populations, re-testing rates among those previously accessing self-tests could be higher than those who have never used an HIVST. Conversely, if never testers are prioritized for HIVST distribution, the HIVST re-testing rates of those who have ever used an HIVST could be lower. There is a paucity of data on re-testing rates in the literature. While direct estimates of rate ratios for repeat testing with HIVSTs are not available, evidence from the STAR and ATLAS initiatives suggests improved testing coverage with HIVST among men and other groups (particularly key populations) who are less likely to engage with traditional testing services (1, 2, 3). A systematic review in Nigeria found a pooled repeat testing rate of 20.1% (95%CI: –11.4% to 51.7%) among HIVST users (4). Similarly, qualitative evidence from South Africa also observed repeat HIV testing practices among past HIVST users, and participants expressed a preference for HIVST as a re-testing approach, even when facility-based testing was available (5). This supports the plausibility that individuals who have previously tested with an HIVST could be more likely to re-test with an HIVST. Thus, we modeled the country-specific *(c)* re-testing rate ratio hierarchically using non-centered parametrization. Specifically, we used a prior mean of 1.2 for the overall re-testing rate ratio with 95% of the prior's density between 0.84 and 1.68.

$$RRoverall^{re-testing} \sim N(logit((1.2 - 0.5)/(2.5 - 0.5)), 0.5)$$

$$RRn^{re-testing}_{c} \sim N(0,1)$$

$$\sigma_{RR^{re-testing}} \sim N(0, 0.5) \quad with \quad \sigma_{RR^{re-testing}} > 0$$

and $RR^{re-testing}_{c} = 0.5 + 2\, logit^{-1}\left(RRoverall^{re-testing} + RRn^{re-testing}_{c}\ \sigma_{RR_{rt}}\right)$

$RRoverall^{re-testing}$: is the overall re-testing rate ratio;

$RRn^{re-testing}_{c}$: is the standard normal variable for country *c*;

$\sigma_{RR^{re-testing}}$: is the scale parameter for $RRn^{re-testing}_{c}$, the between-group standard deviation;

$RR^{re-testing}_{c}$: is the country-specific HIVST re-testing rate ratio.

Finally, it is possible that only a fraction of the distributed HIVST kits are used. Based on findings from the STAR Initiative study in Cameroon reporting an 85% usage rate among kit recipients, the country-specific proportions of HIVST kits being used were modeled hierarchically with the following non-centered parametrization (6).

$$\phi_{overall} \sim N(logit(0.70), 1)$$

$$\phi_{raw} \sim N(0,1)$$

$$\sigma_{\phi} \sim N(0, 0.5) \quad with \quad \sigma_{\phi} > 0$$

$$\phi_c = 0.5 + 0.5\ logit^{-1}(\phi_{overall} + \phi_{raw}\ \sigma_\phi)$$

$\phi_{overall}$: is the overall proportion of distributed HIVST kits that are used;

ϕ_{raw} : is the standard normal variable for country c;

σ_ϕ : is the scale parameter for ϕ_c, the between-group standard deviation;

ϕ_c : country-specific proportion of distributed HIVST kits that are used.

Model outcomes

Using the model, we can calculate the two main outcomes. First is the proportion of the population in country *c*, of sex *j* and in age group *a* that have ever used an HIVST ($p_{c,j,a}(t)$).

$$p_{c,j,a}(t) = \frac{H_{c,j,a}(t)}{H_{c,j,a}(t) + N_{c,j,a}(t)}$$

Second, we defined the equation for the annual total number of HIVST used. If HIVSTs are distributed primarily to certain groups, as opposed to randomly in the population, it could be that using a first HIVST increases the probability of using HIVST in the future. To account for that, we account for a re-testing rate ratio (RR_c^{rt}). Further, since not all distributed tests are being used, we adjust program data on the number of HIVST distributed using a country-specific parameter (ϕ_c). The equation for this outcome is defined as:

$$hivst_c(t) = \int_t^{t+1} \frac{\lambda_{c,j,a}(t)\ (N_{c,j,a}(t) + RR_c^{rt}\ H_{c,j,a}(t))}{\phi_c} \text{ where,}$$

RR_c^{rt}: HIV self-testing re-testing rate ratio for country *c;*

ϕ_c: the country-specific proportion of distributed HIVST kits that are used.

Model likelihood

The observed survey data on the proportion of the population who report ever using an HIVST ($Y_{c,j,a}$) was modelled using a binomial likelihood as follows:

$$Y_{c,j,a}(t) \sim Bin(D_{c,j,a}(t), p_{c,j,a}(t))$$

where,

$Y_{c,j,a}(t)$ = design-adjusted, observed number of individuals having ever used HIVST for country *c*, time t, age group *a* and sex *j;*

$D_{c,j,a}(t)$ = denominator for the effective sample size of the survey for country *c*, sex *j,* age group *a* and time *t;*

$p_{c,j,a}(t)$ = predicted proportion of individuals using HIVST for country *c*, sex *j,* age group *a* and time *t.*

For the HIVST program data, the likelihood follows a normal distribution. Given that the program data does not have any sampling variability, we defined the standard error as 10% of the overall number of HIVST distributed in year *t* to accommodate variations in data accuracy or reporting limitations. The likelihood is given by:

$$hivst_c^{observed}(t) \sim N(hivst_c^{predicted}(t), se_c(t))$$

where,

$hivst_{c,t}^{observed}$ = observed volume of HIVST kits distributed in country *c* and year *t* obtained from program data;

$hivst_{c,t}^{predicted}$ = predicted number of HIVST kits used in country *c* and year *t* (adjusted by ϕ_c);

$se_{c,t}$ = standard error of the observed HIVST data in country *c* and year *t.*

Constraint on the annual number of tests for years without program data

After the last available population surveys, the program data on the annual number of HIVST distributed inform testing rates. Without program data, however, the first-order random walk process for the testing rates quickly leads to very high uncertainty. To reduce the uncertainty, we implemented a constraint on the model-predicted annual number of HIVST distributed to be lower than a certain country-specific threshold (T_c) by penalizing the model's likelihood. This threshold is defined as 4 times the maximum number of observed HIV tests distributed. For countries where the maximum number of HIVST distributed is less than 0.1% of the total size of the 15+ population, we increase the constraint, as defined below:

$$T_c = \begin{cases} 4 \times 0.001 \times pop_c; & if \ \max[hivst_c^{observed}] < 0.001 \times pop_c \\ 4 \times \max[hivst_c^{observed}]; & otherwise \end{cases}$$

where,

pop_c: total population (sex and age aggregated) of a country *c*;

max $[hivst_c^{observed}]$: maximum number of observed HIVST kits distributed among all observed years in country *c.*

We incorporated this as a soft quadratic penalty to the model's log-likelihood in years where program data were not available. This constraint penalizes the country-specific predicted numbers of HIVST distributed that exceed the defined threshold above.

$$P_c(t) = \begin{cases} 0; \ if \ hivst_c^{predicted}(t) \le T_c \\ -\frac{1}{2}\left(\frac{hivst_c^{predicted}(t) - T_c}{\frac{T_c}{4}}\right)^2; if \ hivst_c^{predicted}(t) > T_c \end{cases}$$

And the overall penalty (ψ) as:

$$\psi = \sum_c \sum_t P_c(t)$$

where,

$P_c(t)$: country-specific soft quadratic penalty in the years with missing program data;

$hivst_c(t)$: model predicted annual total number of HIVST used;

T_c: country-specific threshold derived from the maximum number of distributed tests among all observed years.

This results in an overall model log-likelihood of:

$$\mathcal{L}(\theta \mid Y_{c,j,a}(t), hivst_{c,t}^{observed}(t)) = \mathcal{L}(\theta \mid Y_{c,j,a}(t)) + \mathcal{L}(\theta \mid hivst_{c,t}^{observed}(t)) + \psi$$

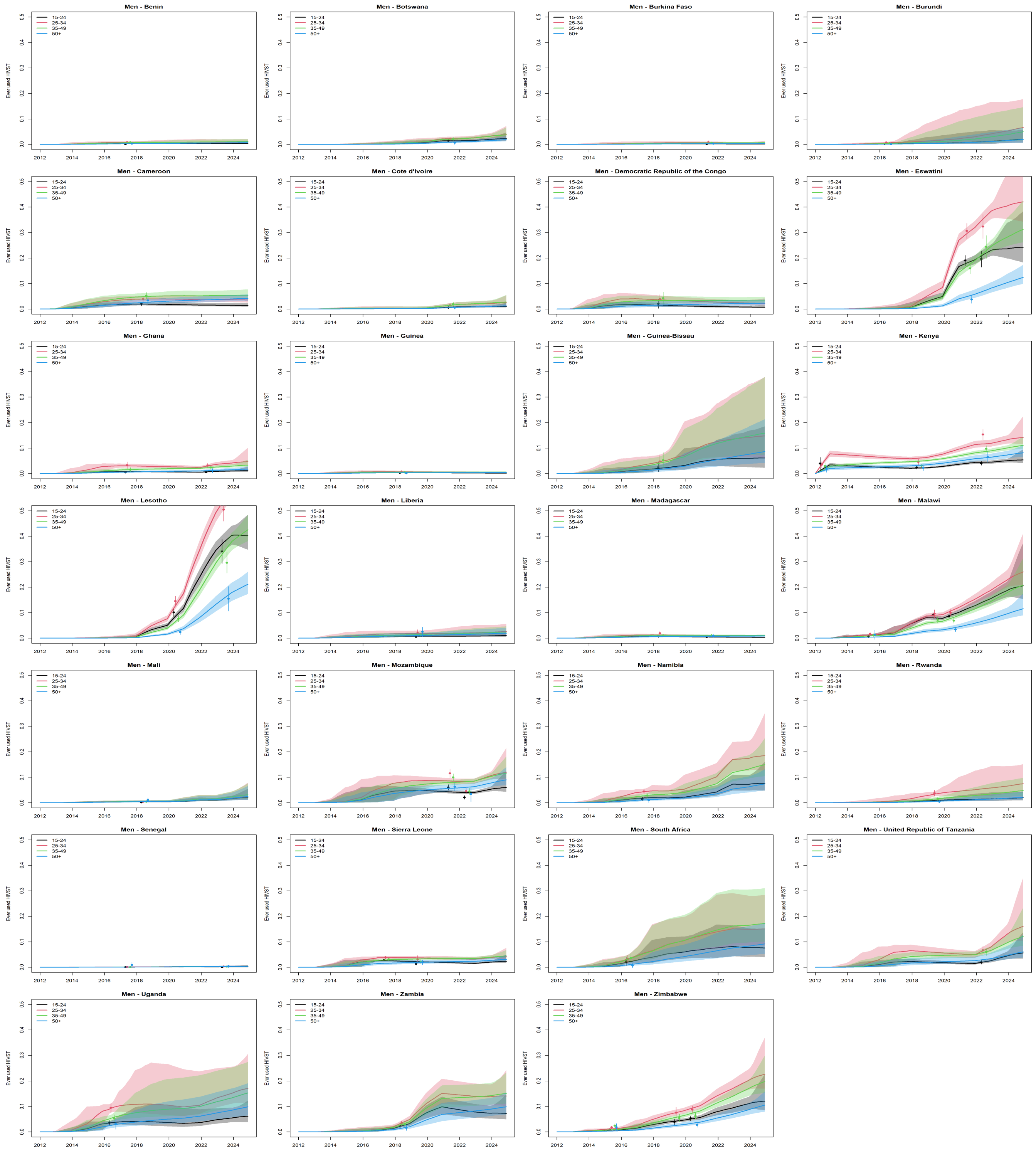

Fig B. Model estimated trends among men by age groups, compared with sex-and-age-stratified survey estimates in 27 African countries (2012-2024). Posterior median estimates (solid lines) and 95% credible intervals (shaded areas) for the proportion of men who have ever used HIVST, shown for each country and stratified by age group (15–24, 25–34, 35–49, 50+ years). Points with error bars represent observed proportions and 95% confidence intervals from surveys.

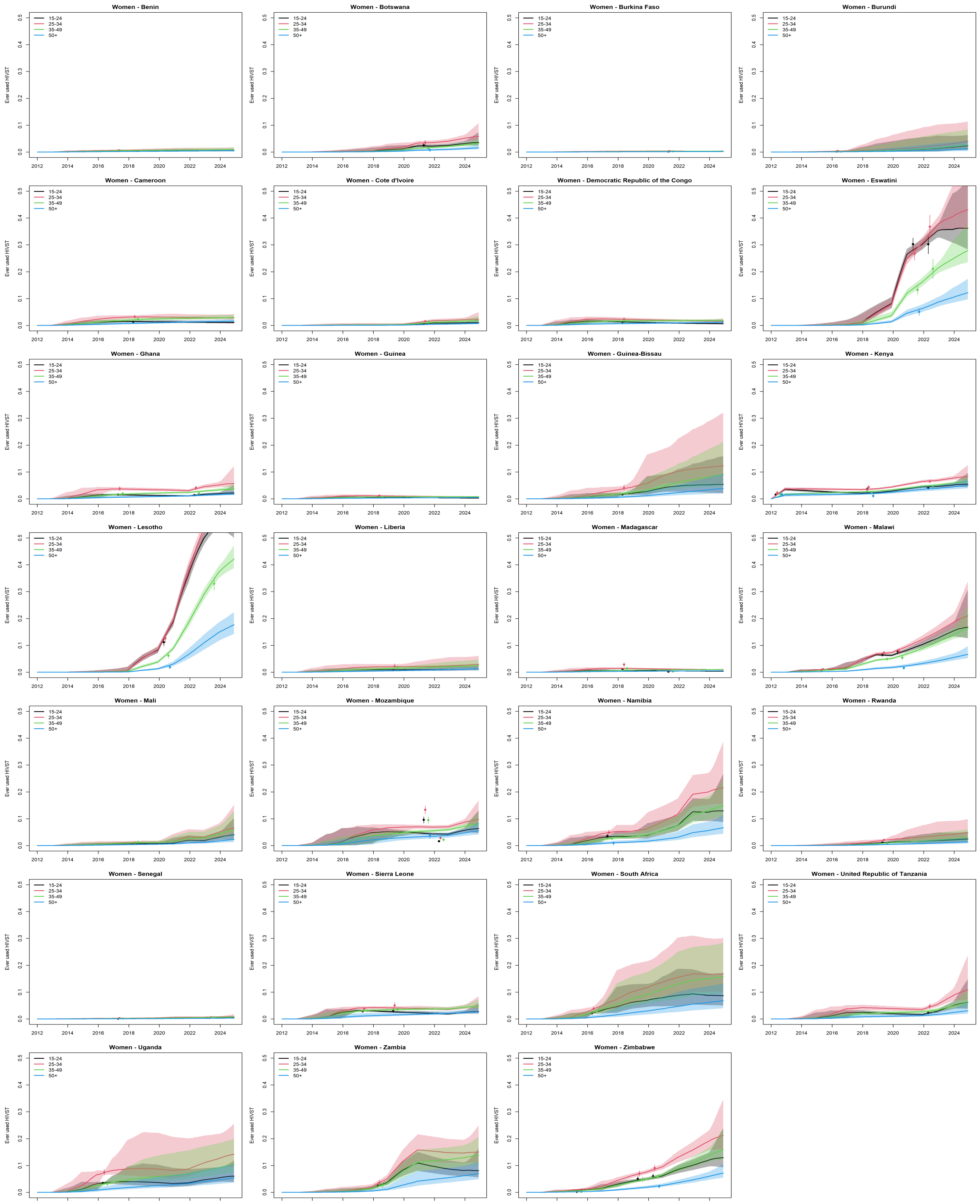

Fig B (continued). Model estimated trends among women by age groups, compared with sex-and-age-stratified survey estimates in 27 African countries (2012-2024). Posterior median estimates (solid lines) and 95% credible intervals (shaded areas) for the proportion of women who have ever used HIVST, shown for each country and stratified by age group (15–24, 25–34, 35–49, 50+ years). Points with error bars represent observed proportions and 95% confidence intervals from surveys.

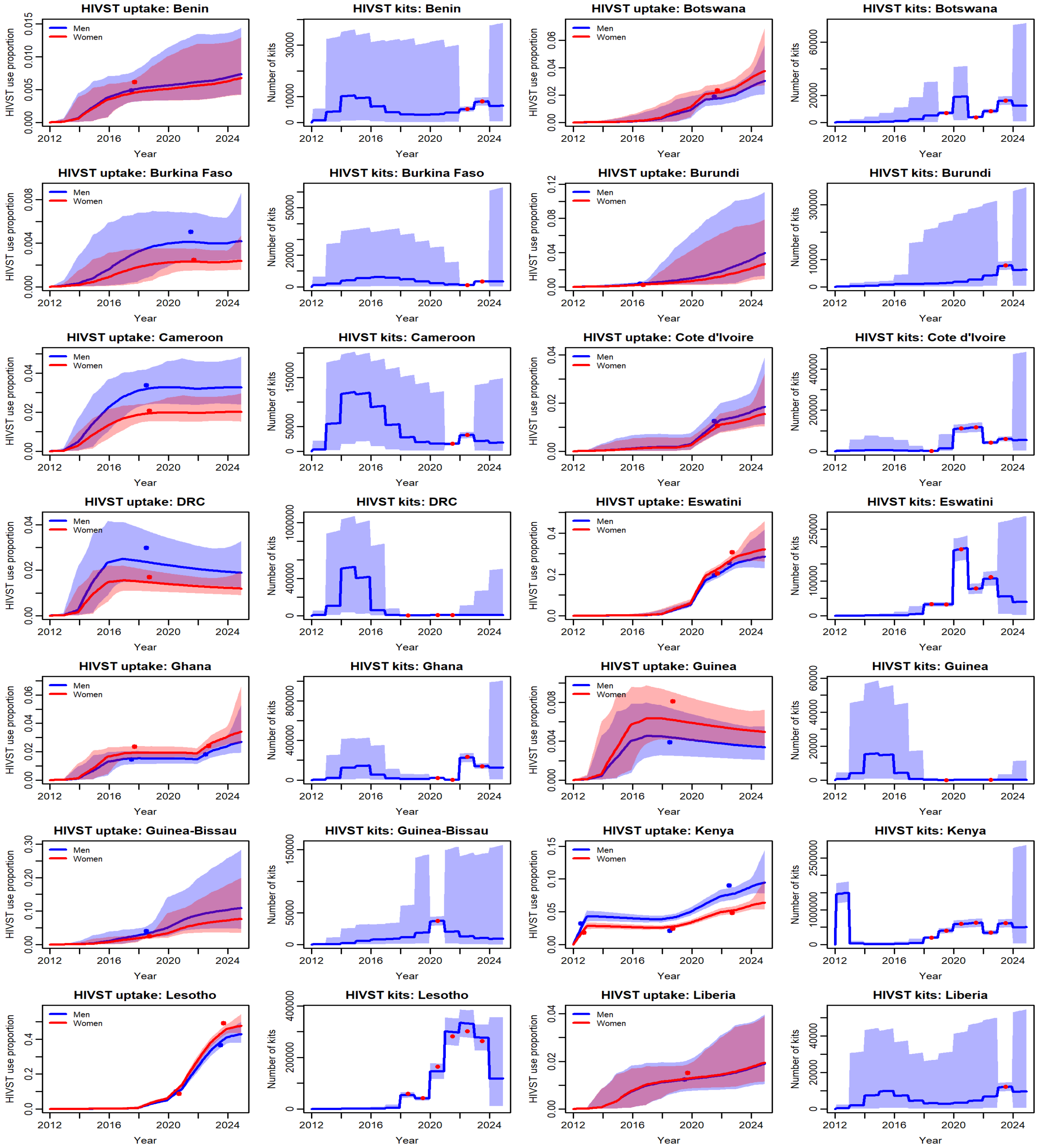

Fig C. Model fits of estimated trends in HIVST uptake by sex (aggregated across age groups) and program data on the number of HIVST kits distributed for 27 African countries (2012-2024). The first columns for each country compare the model estimates aggregated across age groups and stratified by sex (blue for men, red for women). Solid lines and shaded areas respectively represent the posterior medians and 95% credible intervals from the model. The second column for each country shows the corresponding HTS program data on HIVST kit distribution.

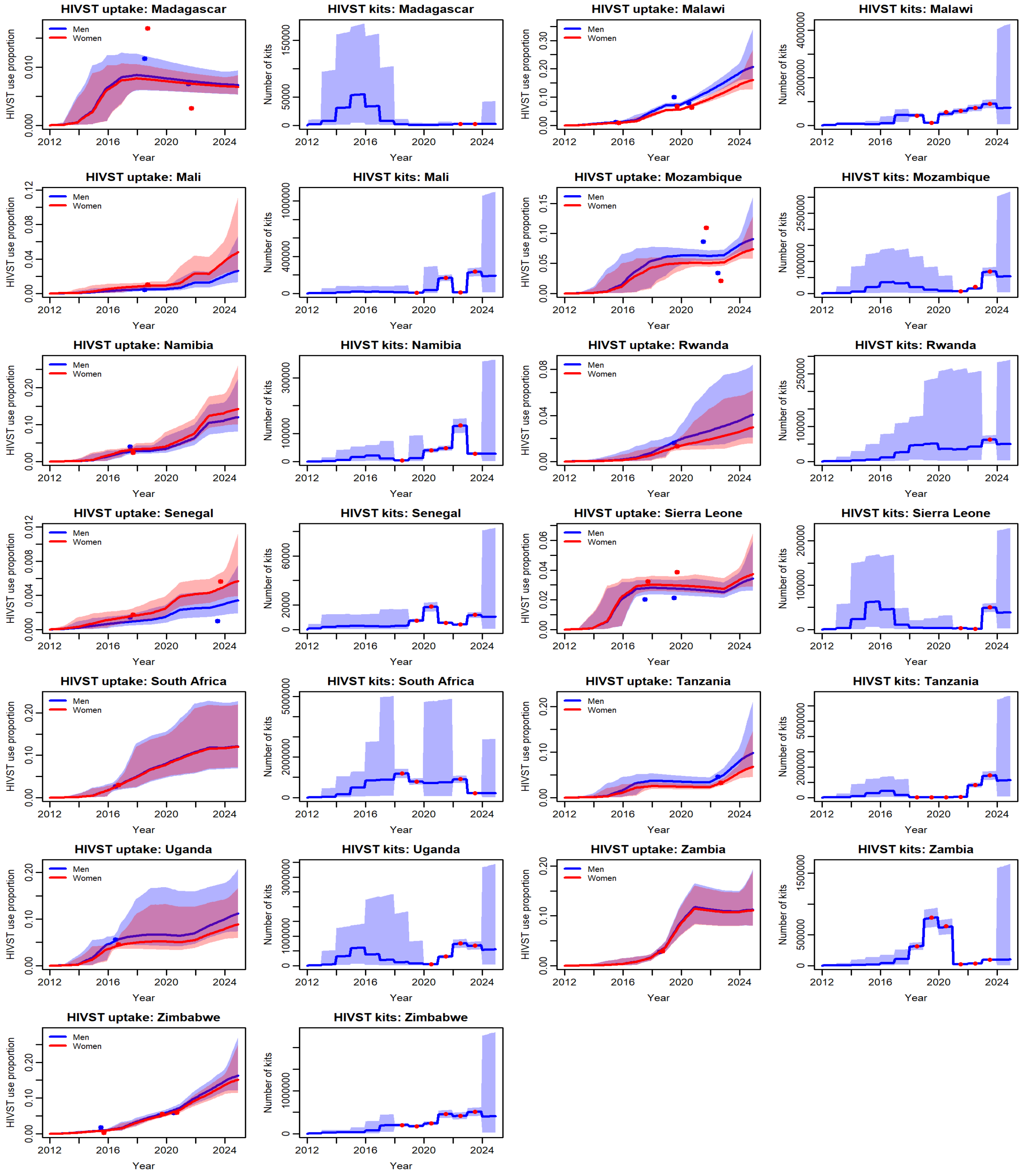

Fig C (continued). Model fits of estimated trends in HIVST uptake by sex (aggregated across age groups) and program data on the number of HIVST kits distributed for 27 African countries (2012-2024). The first columns for each country compare the model estimates aggregated across age groups and stratified by sex (blue for men, red for women). Solid lines and shaded areas respectively represent the posterior medians and 95% credible intervals from the model. The second column for each country shows the corresponding HTS program data on HIVST kit distribution.

Text B. Summary of model convergence and diagnostics

We run 4 chains of 4,000 iterations each (with 2,000 used as warmup). All chains mixed well with no evidence of non-convergence ($\hat{R}$<1.01). Sampling was stable (0 divergent transitions; 0 transitions hit the maximum tree depth). All parameters had an effective sample size (ESS) above 1,947. Visual inspection of trace plots showed good mixing.

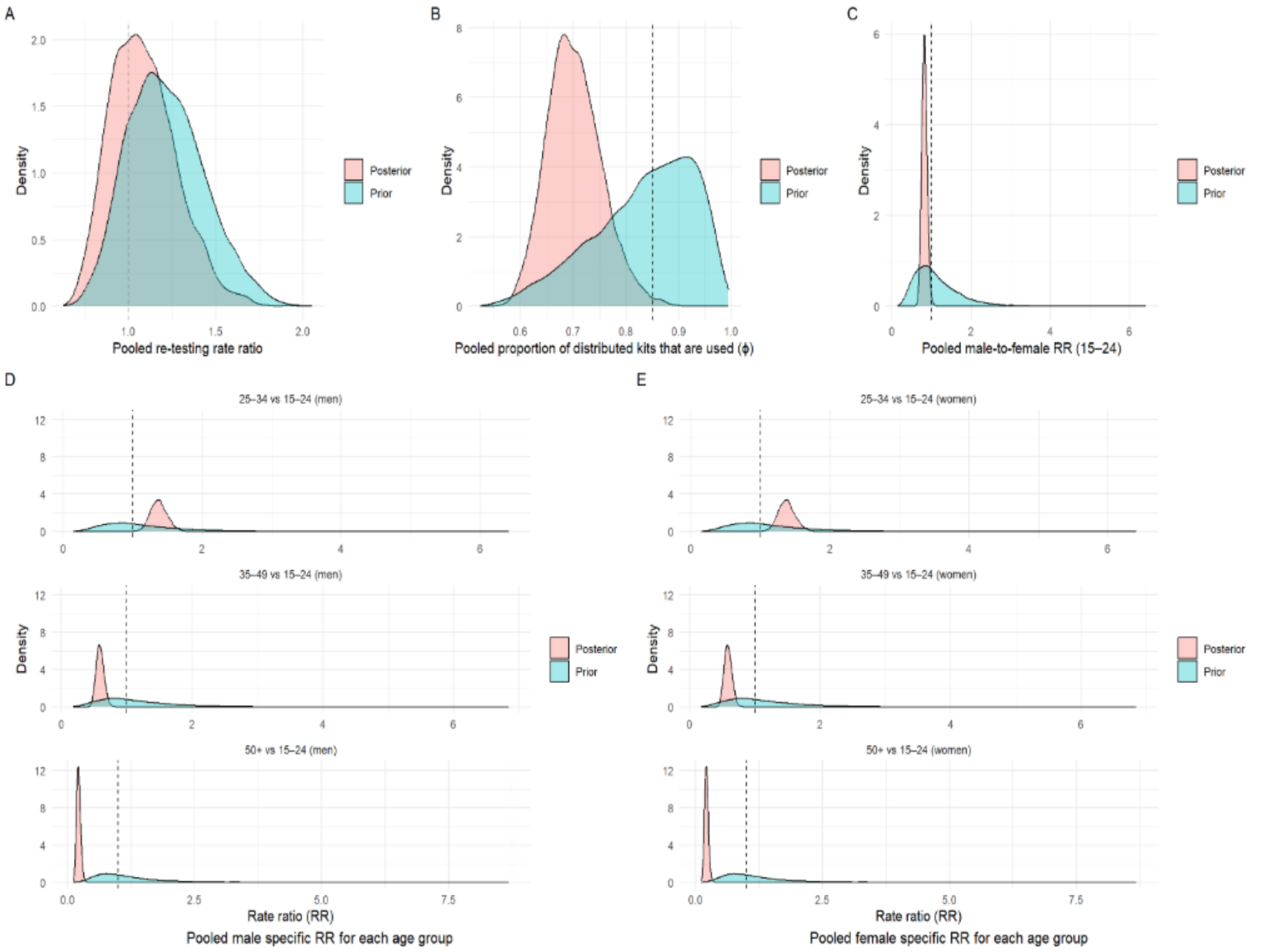

Fig D. Prior-posterior comparison plot for the pooled HIVST re-testing RR (A); pooled proportion of distributed HIVST kits used (B); pooled male-to-female RR for the referent age group (C); pooled male-specific RR for each age group (D); and pooled female-specific RR for each age group (E).

Table E. In-sample comparisons of model fits (overall and by region) with survey and program data.

Region (Number of data points)	Median Error*	Median Absolute Error	% below 2.5th CrI**	% above 97.5th CrI***
**Population-Based Surveys**				
ESA (n=179)	0%-points	0.4%-points	19.0%	20.7%
WCA (n=101)	0%-points	0.1%-points	8.9%	4.9%
Overall (n=280)	0%-points	0.2%-points	15.3%	15.0%
**HIVST Program Data**				
ESA (n=65)	-1.1%	1.1%	0%	1.5%
WCA (n=34)	-1.3%	1.4%	0%	0%
Overall (n=99)	-1.1%	1.2%	0%	1.0%

For survey data, median errors are expressed in percentage points; for HIVST program data, median errors are expressed as percentages relative to observed counts. ESA = Eastern and southern Africa; WCA = Western and central Africa.

*Median error = model-predicted − observed.

**Percentage of observed values below the lower credible interval

***Percentage of observed values above the upper credible interval

Table F. Number of parameters estimated in the HIV self-testing model.

Parameter categories	Dimension	Number parameters
Country-year baseline testing rates	27 countries × 13 years	351
Standard deviation hyperparameters	6	6
Overall pooled hyperparameters	1 + 1 + 1 + 3 + 3	9
Country-specific re-testing rates	27 countries	27
Country-specific proportion of distributed kits used	27 countries	27
Country-specific 15-24-year-old male-to-female rate ratios	27 countries	27
Country-specific male RRs for each age group	3 × 27 countries	81
Country-specific female RRs for each age group	3 × 27 countries	81
**Total**		**609**

References

1. STAR Initiative. Knowing your status—then and now: Realizing the potential of HIV self-testing. Geneva; 2018.

2. Hatzold K, Gudukeya S, Mutseta MN, Chilongosi R, Nalubamba M, Nkhoma C, et al. HIV self-testing: breaking the barriers to uptake of testing among men and adolescents in sub-Saharan Africa, experiences from STAR demonstration projects in Malawi, Zambia and Zimbabwe. Journal of the International AIDS Society. 2019;22:e25244.

3. Rouveau N, Ky-Zerbo O, Boye S, Fotso AS, d'Elbée M, Maheu-Giroux M, et al. Describing, analysing and understanding the effects of the introduction of HIV self-testing in West Africa through the ATLAS programme in Côte d'Ivoire, Mali and Senegal. BMC Public Health. 2021;21:1-14.

4. Eleje GU, Emmanuel GO, Akinsolu FT, Foláyan MO. Assessment of the acceptability and detection rate of HIV self-testing in Nigeria: a systematic review and meta-analysis. Discover Epidemics. 2024;1(1):1-18.

5. Harichund C, Kunene P, Simelane S, Abdool Karim Q, Moshabela M. Repeat HIV testing practices in the era of HIV self-testing among adults in KwaZulu-Natal, South Africa. PloS one. 2019;14(2):e0212343.

6. Bouba Y, Djomo ARD, Mouliom FN, Souleymanou A, Lifanda E, Liman Y, et al. Evaluating the effectiveness of oral HIV self testing according to distribution models in Cameroon. Scientific Reports. 2024;14(1):30694.
